# Smart Nanoparticles for Selective Immobilization of Acid Phosphatases

**DOI:** 10.1002/cctc.201800405

**Published:** 2018-07-17

**Authors:** Flóra Nagy, Gábor Tasnádi, Diána Balogh‐Weiser, Evelin Bell, Mélanie Hall, Kurt Faber, László Poppe

**Affiliations:** ^1^ Department of Organic Chemistry and Technology Budapest University of Technology and Economics Műegyetem rkp. 3 1111 Budapest Hungary; ^2^ Austrian Centre of Industrial Biotechnology,*c*/*o*Department of Chemistry, Organic & Bioorganic Chemistry University of Graz Heinrichstrasse 28 8010 Graz Austria; ^3^ Department of Chemistry, Organic & Bioorganic Chemistry University of Graz Heinrichstrasse 28 8010 Graz Austria; ^4^ Biocatalysis and Biotransformation Research Center Faculty of Chemistry and Chemical Engineering Babes-Bolyai University of Cluj-Napoca Arany János str. 11 400028 Cluj-Napoca Romania

**Keywords:** continuous flow synthesis, selective enzyme immobilization, silica nanoparticles, surface modification, transphosphorylation

## Abstract

An easy to use method combining the selectivity of metal chelate affinity binding with strong covalent linking was developed for immobilization of non‐specific acid phosphatases bearing a His‐tag from crude cell lysate. Silica nanoparticles were grafted with aminopropyl functions which were partially transformed further with EDTA dianhydride to chelators. The heterofunctionalized nanoparticles charged with Ni^2+^ as the most appropriate metal ion were applied as support. First, the His‐tagged phosphatases were selectively bound to the metal‐chelate functions of the support. Then, the enzyme‐charged silica nanoparticles were further stabilized by forming a covalent linkage between nucleophilic moieties at the enzyme surface and free amino groups of the support using neopentylglycol diglycidylether as the most effective bifunctional linking agent. The phosphatase biocatalysts obtained by this method exhibited better phosphate transfer activity with a range of alcohols and PP_i_ as phosphate donor in aqueous medium applying batch and continuous‐flow modes than the ones immobilized on conventional supports. Furthermore, this novel strategy opens up novel possibility for efficient immobilization of other His‐tagged recombinant enzymes.

## Introduction

Phosphorylation is a key reaction in biology involved in a broad range of vital processes, such as cell signal transduction, nucleic acid synthesis and energy transfer,^1^ while medicinal chemistry makes use of phosphate monoesters as prodrugs.^2^,^3^ Synthetic chemistry requires activated phosphate precursors (usually in form of toxic chemicals) to form phosphate esters. Although mild metal‐catalyzed and organocatalytic methods have been recently disclosed,^4^ chemo‐ and regio‐selectivity remains a challenge, moreover, the products are typically phosphate triesters requiring further deprotection to yield monoesters. In biological systems, phosphorylation is carried out by kinases depending on ATP as a phosphate donor. The disadvantage of kinases lies in their narrow substrate scope^5^, ^6^, ^7^ and the modest efficiency of ATP‐recycling (several 100s at best), which limits their applications on preparative‐scale.^8^,^9^

Alternatively, ATP‐dependent kinases could be rivaled by phosphatases (phosphate monoester hydrolases), which can employ inexpensive inorganic polyphosphates [e. g. pyrophosphate (PP_i_), triphosphate (PPP_i_), or polyphosphoric acid (polyP)] as phosphate donor.^10^,^11^ Phosphatases generally exhibit a broad substrate scope as a result of their involvement in biodegradation pathways. However, undesired product hydrolysis (originating from the natural hydrolytic activity) renders their practical application in synthesis challenging. Non‐specific acid phosphatases from *Shigella flexneri* (PhoN‐Sf) and from *Salmonella enterica* ser. *typhimurium* LT2 (PhoN‐Se) chemo‐ and regiospecifically phosphorylate a wide range of monoalcohols, diols and sugars with remarkably stable turnover^12^,^13^ on gram‐scale.^14^, ^15^, ^16^, ^17^, ^18^ Using an immobilized phosphatase in a continuous‐flow packed‐bed reactor resulted in kinetically controlled transphosphorylation and accumulation of the phosphorylated product.^16^,^17^,^19^ In addition, enzyme immobilization could exhibit various advantages, such as the ease of separation from the reaction mixture, reusability, and an increase in stability and organic solvent tolerance.^20^

Several techniques are available for protein immobilization, such as adsorption, covalent attachment onto solid supports, cross‐linking and entrapment in matrices.^21^ The choice of immobilization method is key to retain optimal catalytic activity. In contrast to entrapped enzymes, where diffusion is often limited, immobilization of enzymes onto supports with high surface area ensures easy access of the substrate to the protein indicating the critical role of choosing the right type of support. An ideal support displays hydrophilicity, biocompatibility, resistance to microbial attack and to physical compression and is available at low cost.^22^,^23^ Silica‐based supports are one of the most suitable matrices for enzyme immobilization in industry,^24^,^25^ and among them, silica nanoparticles (SNPs) are characterized by their high surface to volume ratio, mass transfer rates and good enzyme loading capacity.^26^ Moreover, preparation of SNPs via hydrolysis‐condensation of low‐priced precursors, such as tetraethyl orthosilicate mediated by acid or base catalysts is straightforward, while modification of their surface is enabled by various organosilane precursors. Enzyme immobilization on nanoparticles can reduce protein unfolding and improve operational stability.^27^ In addition, biocompatibility of SNPs is assured and no acute toxicity is currently known.

Immobilization from crude cell lysate overcomes time‐consuming purification often accompanied by enzyme inactivation. Immobilized metal‐chelate affinity chromatography (IMAC) is a well‐developed tool for pilot‐scale purification of poly‐His tagged proteins.^28^, ^29^, ^30^, ^31^, ^32^ Due to the reversibility of the binding process resulting in undesired release of the enzyme and/or the metal to the reaction medium, application of metal‐chelate affinity methods to produce supported biocatalysts was only recently considered.^33^,^34^ By proper choice of the chelating metal ion, stable biocatalyst preparations could be obtained.^35^

Alternatively, covalent bonds could be formed upon selective adsorption of the target protein onto heterofunctionalized epoxy‐supports.^36^, ^37^, ^38^ The epoxy functions can react with different nucleophilic groups (such as amine, thiol or carboxylate) on the protein surface under mild conditions and these advantageous properties led to the idea of using bisepoxides for enzyme immobilization.^39^, ^40^, ^41^, ^42^ Thus, poly(ethylene‐glycol)‐diglycidyl ether was applied for binding various oxidases onto a biosensor microelectrode^.[43]^. Glycerol diglycidyl ether was used as cross‐linking agent for immobilization of lipases and phenylalanine ammonia‐lyase (PAL) yielding cross‐linked enzyme aggregates (CLEAs)^44^ and for binding of PAL^45^ or *Candida antarctica* lipase B (CALB)^46^ onto carbon nanotubes. Later, a method was developed for immobilization of CALB47a and ω‐transaminase from *Chromobacterium violaceum* 47b on bisepoxide‐activated mesoporous aminoalkyl polymer supports.

**Figure 1:**
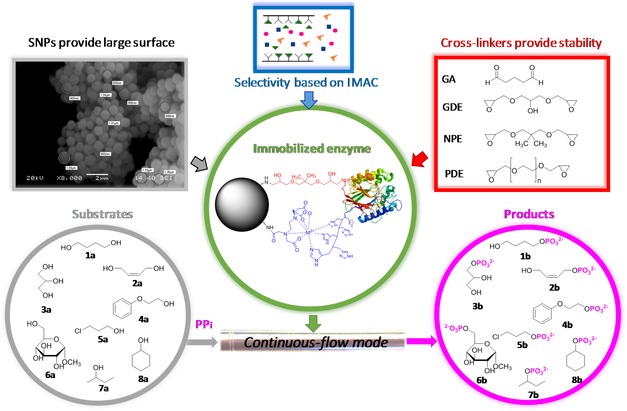
A novel method using alkylamino‐/metal‐chelate‐grafted SNPs and bisepoxide covalent linkers to immobilize non‐specific acid phosphatases for regioselective transphosphorylation of alcohols **1 a**–**8 a** (SNPs: silica nanoparticles; IMAC: immobilized metal‐chelate affinity chromatography; PP_i_: pyrophosphate; GA: glutaraldehyde; GDE: glycerol diglycidyl ether; NPE: neopentylglycol diglycidyl ether; PDE: polyethylene glycol diglycidyl ether).

In this study, a heterofunctionalized aminopropyl‐chelate silica nanoparticle support combining the advantages of SNPs, the IMAC protein enrichment and covalent linking of proteins with bisepoxides was developed and applied for selective immobilization of poly‐His‐tagged acid phosphatases as model proteins (Figure 1). The immobilized biocatalysts favorably compared with preparations using commercial supports (Immobead 150, Relizyme HA403/M, EziG™ 2Fe) in a fed‐batch‐ or a packed‐bed continuous flow reactor using PP_i_ as phosphate donor.

## Results and Discussion

### Synthesis and Surface Modification of Silica Nanoparticles

The so‐called “Stöber method” is an excellent process to prepare monodispersed silica particles with a diameter ranging from ∼50 nm to a few μm.^48^ By optimizing the key parameters, i. e. concentration of tetraethyl orthosilicate (TEOS) and ammonia, solvent and time, high yield of nanosized particles can be obtained. Hydrolysis and condensation of TEOS in 1‐butanol at high concentration of NH_3_ gave particles close to micron‐size for application in a packed‐bed reactor for continuous flow synthesis of phosphorylated compounds. Scanning electron microscopy (SEM) was used to analyze the morphology and SEM micrographs shown in Figure S4 indicated monodispersed and spherical SNPs of 950–1000 nm.

Surface grafting of SNPs was performed with 3‐aminopropyltrimethoxysilane (APTMS) to obtain aminopropyl‐functionalized silica nanoparticles (ASNPs) to provide suitable functions to attach various molecules to surfaces (Figure S5).^32^

Using appropriate amounts of APTMS during surface grafting, aggregation was prevented by using ethanol as solvent (Figure S5). Next, partial functionalization of the aminopropyl residues on the surface was performed using EDTA dianhydride resulting in a mixed alkylamino‐/metal‐chelator‐grafted silica support which could chelate metal ions for protein fixation. Since the metals used in IMAC exhibited altered affinities towards different proteins, Ni^2+^, Co^2+^, Cu^2+^, Fe^3+^, Zn^2+^ and La^3+^ were tested for phosphatase binding.

**Figure 2:**
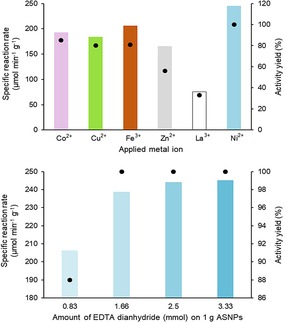
Activity yield (dots) and specific reaction rate (*r*_batch_) (bars) of PhoN‐Sf attached onto heterofunctionalized ASNPs, (A) as a function of metal ions and (B) as a function of the amount of EDTA dianhydride during functionalization of ASNP (1 g, tested after Ni^2+^ charging). In each case, PhoN‐Sf (1 U) was immobilized on functionalized dry ASNP (1 mg). Activity yield is expressed as the percentage of total activity (U) immobilized on the support related to the initial activity (determined by the *p*NPP assay).

In the first binding tests, a cell‐free extract of His‐tagged PhoN‐Sf (10 U of lysate, corresponding to 0.833 mg total protein) was adsorbed on the metal chelate supports (10 mg). Gel electrophoresis (Figure S7–8) showed good immobilization selectivity in the case of Ni^2+^ and Zn^2+^, however, the latter exhibited weaker protein binding capacity (Figure 2A).

The activity of immobilized PhoN‐Sf on heterofunctionalized metal‐chelate supports was verified by hydrolysis of 4‐nitrophenyl phosphate (*p*NPP) using a spectrophotometric assay.^16^ Figure 2A shows the specific reaction rate (*r*_batch_)^49^ and the activity yield (*Y*_A_) of the immobilized PhoN‐Sf biocatalysts depending on the metal employed (for definitions of *r*_batch_ and *Y*_A_, see Experimental section). Because immobilization was carried out from fermentations crude lysate, it was important to determine how many target proteins (enzymes) were bound to the carrier.

The Ni^2+^‐chelating support could bind 100 % of the loaded phosphatase activity and exhibited the highest *r*_batch_ (245.1 U g_biocatalyst_^−1^) and hence was selected for further studies.

Mateo *et al*. reported that the use of the metal charged chelator at low density (5‐10 μmol/mL Co^2+^ or Cu^2+^ ions) increased the selectivity of the adsorption.^38^ We observed that during the heterofunctionalization of the aminopropylated SNPs 1.66 mmol g^−1^_SNP_ of EDTA dianhydride was sufficient to achieve high *r*_batch_ after enzyme loading (Figure 2B), therefore this reagent amount was selected for the further studies to leave sufficient fraction of free amino groups on the surface for subsequent covalent linking.

**Figure 3:**
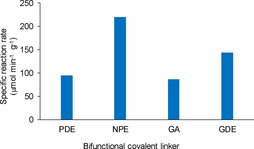
Specific reaction rate (*r*_batch_) of immobilized PhoN‐Sf biocatalysts [1 U PhoN‐Sf was applied to 1 mg of ASNP‐E‐Ni followed by addition of the indicated bifunctional covalent linkers (GA: glutaraldehyde; GDE: glycerol diglycidyl ether; NPE: neopentylglycol diglycidyl ether; PDE: polyethylenglycol diglycidyl ether)].

Covalent linkage between the protein and the amino groups of the carrier surface can contribute remarkably to the stabilization of the immobilized biocatalyst. Hence, three bisepoxides with different lengths and hydrophobicities and glutaraldehyde (GA) were selected as bifunctional covalent linking agents (Figures 1, 3).

Because of the poor water solubility of bisepoxides, 20 v/v % of organic cosolvent (EtOH) was used during covalent linking. As shown in Figure 3, PhoN‐Sf on ASNP‐E‐Ni treated with neopentylglycol diglycidyl ether retained ∼90 % of its activity during covalent linking (219.5 U g_bioctalyst_^−1^), while the other preparations suffered significant losses. This rationalized the use of neopentylglycol diglycidyl ether as cross‐linker in the further studies (for abbreviations of the functionalized SNPs and the novel biocatalysts in the further studies, see Table 1).

**Table 1:** Immobilization of acid phosphatases by selective binding on aminopropyl silica nanoparticles (ASNPs) partially functionalized with an EDTA‐dianhydride derived chelator (ASNP‐E)^[a]^ and impregnated with different metal ions (ASNP‐E‐M)^[b]^ followed by post cross‐linking^[c]^ (Figure S3).

Enzyme	Optimal Biocatalyst
PhoN‐Sf	ASNP‐E‐Ni/NPE/PhoN‐Sf
PhoN‐Se	ASNP‐E‐Ni/NPE/PhoN‐Se

[a] The optimal amount of EDTA‐dianhydride was 2.5 mmol/g ASNPs, see Figure 2B. [b] The optimal metal ion (M^n+^) for selective binding was Ni^2+^, see Figure 2A. [c] The optimal bifunctional cross‐linking agent was neopentylglycol diglycidyl ether (NPE), see Figure 3.

**Figure 4:**
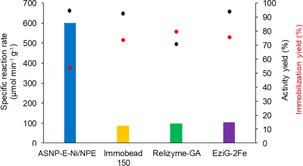
Activity yield (*Y*_A_: •), immobilization yield (*Y*_I_: •) and specific reaction rate (*r*_batch_: colored bars) of PhoN‐Sf immobilized onto various biocatalysts (2 U of PhoN‐Sf was immobilized onto 1 mg of dry support). 3 mg immobilized biocatalyst was used in the reaction mixture.

Since the activity yield of immobilized PhoN‐Sf was 100 % in the case of ASNP‐E‐Ni/NPE as support (Figure 2), indicating incomplete protein loading, the amount of protein was doubled (2 U mg^−1^ carrier in all cases of supports), which led to a dramatic increase of the *r*_batch_ (Figure 4, ∼220 to ∼600 U g_bioctalyst_^−1^), maintaining almost 100 % activity yield. The main reasons of the increased specific activity are twofold: I) the support binds the target enzyme selectively even from the lysate and II) the average pore diameter between the rigid enzyme‐coated SNPs is large ∼200‐400 nm (see Figure S4–S6) in the smaller ASNP‐E‐Ni/NPE/PhoN‐Sf particles reducing significantly the mass transfer limitations of substrate influx and product efflux which occur in porous supports. SDS PAGE analysis of crude lysate of PhoN‐Sf compared with ASNP‐E‐Ni/NPE/PhoN‐Sf (Figure S7) indicated 50 % enzyme content in the crude lysate proteins.

### Characterization of the Functionalized ASNPs and the Immobilized ASNP‐E‐Ni/NPE/PhoN‐Sf Biocatalyst

The amino group content on the preparations was determined by ninhydrin colorimetric assay (see in Table S1) revealing 386 mol g^−1^ for the aminopropyl‐grafted silica nanoparticles (ASNPs) 113 mol g^−1^ for the nanoparticles after partial derivatization with EDTA‐dianhydride (ASNP‐E). Consequently, ∼70 % of the original amine content of ASNPs was functionalized with EDTA‐dianhydride and a residual 30 % of the original amine groups remained available on ASNP‐E for further covalent functionalization with bifunctional cross‐linking agent.

Elemental analysis on the surface of the nanoparticles was carried out with energy dispersive spectroscopy/energy dispersive X‐ray analysis [EDS/EDAX with Si(Li) detector; see Figure S2 and Table S2]. Analysis of the chelating nanoparticles (ASNP‐E) impregnated with two different metal ions (Ni^2+^ and Co^2+^) resulted in an interesting outcome. After impregnation of ASNP‐E with Co^2+^ ions, EDS/EDAX elemental analysis revealed 1.7 % Co besides 4.28 % C and 1.10 % N, while after impregnation with Ni^2+^ ions, 0.15 % of Ni, besides 4.43 % C and 1.21 % N could be determined on the surface of ASNP‐E/M supports. Based on the results of the ninhydrin assay revealing 30 % residual amine group content on ASNP‐E and assuming 1 : 1 chelation with a metal ion M^2+^, a relative elemental abundance of 10C+2.7 N+0.7 M [i. e. 0.7×(13C+3N+1M)+0.3×(3C+1N)] could be calculated. The found relative elemental abundance of 10C+2.7N+0.34Ni revealed that, as an average, two EDTA‐dianhydride derived chelator units complexed one Ni^2+^. On the other hand, the measured relative elemental abundance of 10C+2.7N+3.8Co after impregnation with Co^2+^ indicated that the Co^2+^ could be present mostly in other than chelated state on the surface of nanoparticles (0.7 part of Co in 1 : 1 chelation, an additional 3.1 part of Co in other state). These result could account for the lower efficiency of ASNP‐E‐Co nanoparticles in the final immobilization of PhoN‐Sf.

An elemental ratio for His_6_‐PhoN‐Sf could be calculated by using its sequence (UniProt: Q7BEK9) complemented with His_6_‐tag (C_1244_H_1937_N_351_O_377_S_6_, i. e. 3.54C for 1.00N). On the surface of the ASNP‐E‐Ni/NPE/PhoN‐Sf biocatalyst, the relative elemental abundance measurement indicated 23.96 % C and 1.88 % N [12.74C to 1.00N, which corresponds to (9.2+3.54)C for 1.00N]. Because only the enzyme contained N but the cross‐linking NPE not, ∼28 % of the C content originated from the enzyme and ∼72 % of the C content belonged to the cross‐linker. This massive covalent multipoint binding and cross‐linking could account for the improved mechanical properties of the preparation and also for the enhanced stability of the enzyme in transphosphorylation.

### Comparison of ASNP‐E‐Ni/NPE/PhoN‐Sf with PhoN‐Sf Biocatalysts on Commercial Supports

The designed mode of immobilization was based on IMAC principle and epoxy‐chemistry for covalent binding. Therefore, our novel immobilization method was compared to commercial carriers using these principles for immobilization [Immobead 150: an epoxy resin for covalent binding; three EziG^TM^‐Fe supports: Fe‐ion‐chelator‐based carriers for selective immobilization of His‐tagged proteins (see Table S5) and Relizyme‐GA: an aminoalkylated polyacrylate resin activated with glutaraldehyde for covalent binding] (see Table S6). Although it was obvious that the pH required by the acid phosphatases during the transphosphorylation reactions (pH 4.2) was out of the recommended working range of EziG^TM^‐Fe supports (pH 5–10), the PhoN‐Sf biocatalyst on EziG^TM^‐2Fe was included in the comparison as an example of IMAC‐principle based commercial solution enabling selective immobilization of His‐tagged proteins. Moreover, immobilization and activity determinations by *p*NPP assay for EziG^TM^‐2Fe/PhoNs were carried out at pH values (7.5 and 6, respectively) which are within the recommended working pH range.

Figure 4 shows that the *r*_batch_ of immobilized ASNP‐E‐Ni/NPE/PhoN‐Sf was significantly higher than that of obtained with commercial supports (∼600 U mg^−1^ vs.; ∼80–100 U mg^−1^, see Table S2 and Table S6 for comparison). Activity yields were largely comparable (>90 %, except the 70 % for Relizyme‐GA/PhoN‐Sf) and immobilization yields were 74–80 % in the case of commercially available supports, while the lower 54 % value for ASNP‐E‐Ni/NPE/PhoN‐Sf (see Figure 4) was mostly due to the selective protein binding.

### Immobilized PhoN‐Sf in Transphosphorylation Reactions

**Figure 5:**
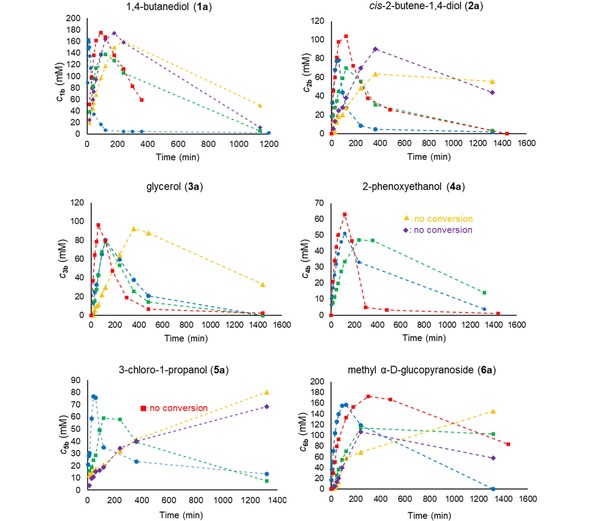
Time course profile of the product formation during phosphorylation of 1,4‐butanediol (**1 a**, 500 mM), *cis*‐2‐butene‐1,4‐diol (**2 a**, 300 mM), glycerol (**3 a**, 500 mM), 2‐phenoxyethanol (**4 a**, 100 mM), 3‐chloro‐1‐propanol (**5 a**, 500 mM) and methyl‐α‐d‐glucopyranoside (**6 a**, 500 mM) in shake vials using PhoN‐Sf immobilized by various methods. Reaction conditions: PP_i_ (250 mM), PhoN‐Sf biocatalysts (3 mg, immobilized; or 6 U, 500 μg as lysate), DMSO (1 %, as internal standard), in 1 mL final volume, pH 4.2, 30 °C, 750 rpm shaking. Data series represent product concentrations with PhoN‐Sf biocatalysts on ASNP‐E‐Ni/NPE (•), Relizyme‐GA (▪), Immobead 150 (▴) or (⧫) EziG^TM^‐2Fe, or with PhoN‐Sf lysate (▪).

In order to test the activity of the immobilized phosphatases in transphosphorylation mode (which does not necessarily correlate with those obtained in the pNPP hydrolytic assay), batch reactions were performed with 1,4‐butanediol (**1 a**) as model substrate using PP_i_ (250 mM) as phosphate donor at pH 4.2. Initial formation of phosphorylated product was always followed by hydrolytic degradation of the formed product in the batch systems. Formation of **1 b** catalyzed by the soluble lysate (6 U, 500 μg protein) or immobilized PhoN‐Sf biocatalysts (3 mg) is shown on Figure 5. The different immobilization methods influenced significantly the maximal product concentration and the reaction rate. ASNP‐E‐Ni/NPE/PhoN‐Sf exhibited the highest reaction rate reaching the maximal product amount (163 mM) within only 9 min. Although similar or slightly higher product levels were observed with PhoN‐Sf lysate, with Immobead 150/PhoN‐Sf or with EziG^TM^‐2Fe/PhoN‐Sf, the time needed to reach maximum production was significantly longer (175 mM, 90 min, 160 mM, 240 min and 174 mM, 180 min, resp.). In contrast, Relizyme‐GA/PhoN‐Sf furnished reduced product levels after 90 min (138 mM). Although product hydrolysis rates were also higher than that of the commercial supports and lysate, the high space‐time yield (STY) obtained with ASNP‐E‐Ni/NPE/PhoN‐Sf (183 g L^−1^ h^−1^ vs. 7–20 g L^−1^ h^−1^) could be advantageous in continuous flow mode owing to reduced residence time. The improved reaction rate obtained with ASNP‐E‐Ni/NPE/PhoN‐Sf is presumably due to its high surface area and a higher selectivity of the immobilization protocol, as this support binds a maximum of phosphatase but less host proteins.

To further evaluate the general applicability of ASNP‐E‐Ni/NPE/PhoN‐Sf, substrates **2 a**–**6 a** were tested (Figure 5). The performance of soluble PhoN‐Sf as lysate containing the same amounts of units as subjected to the immobilization process were also performed with all the substrates in batch experiments (Figure 5, Table 2). Comparisons between the productivity of the soluble and immobilized forms of the enzyme using their *r*_batch_ values was at the same degree of conversions are shown in Table 2. The results clearly showed that in most cases the catalytic properties of the immobilized biocatalysts were improved. Only in the case of **2 a** and **3 a** was the specific activity of the soluble enzyme slightly higher than that of ASNP‐E‐Ni/NPE/PhoN‐Sf. Moreover, compound **5 a**, which could be transphosphorylated with the soluble enzyme below 300 mM concentration,^16^ was not transformed by the lysate at 500 mM concentration, presumably due to substrate inhibition. Importantly, the immobilized PhoN‐Sf forms could tolerate this high substrate concentration.

**Table 2:** Comparison of the specific reaction rates of transphosphorylation reactions with soluble and immobilized PhoN‐Sf in batch mode at the same level of conversions laying in the linear phase of the reactions.

	Soluble PhoN‐Sf ^[a^			ASNP‐E‐Ni/NPE/PhoN‐Sf ^[b]^		
Substrate	*Time* [min]	*c* [%]	*r*_batch_ [U/mg]	*Time* [min]	*c* [%]	*r*_batch_ [U/mg]
**1 a**	30	18.3	13.1	9	16.4	43.5
**2 a**	30	21.5	10.8	30	22.5	10.0
**3 a**	15	5.1	7.5	30	4.8	3.7
**4 a**	15	18.5	4.7	15	16.1	5.0
**6 a**	60	20.3	6.5	30	19.6	15.5

[a] Amount of soluble PhoN‐Sf the was 0.25 mg (assuming 50 % PhoN‐Sf content in the lysate; see SDS gel picture Figure S7). [b] Amount of enzyme in ASNP‐E‐Ni/NPE/PhoN‐Sf was 0.225 mg on 3 mg beads (as *Y*_A_=90 %).

In comparison with other immobilized PhoN‐Sf forms, ASNP‐E‐Ni/NPE/PhoN‐Sf delivered product levels similar to that obtained with PhoN‐Sf on commercial carriers but within significantly shorter reaction time resulting in superior STYs (see Table S6). Among the commercial supports, Relizyme‐GA/PhoN‐Sf delivered the highest reaction rates, which is in good accordance with previous observations,^16^,^17^ on Immobead 150/PhoN‐Sf and EziG^TM^‐2Fe/PhoN‐Sf displayed similar (slower) reaction profiles in transphosphorylation and hydrolysis compared to ASNP‐E‐Ni/NPE/PhoN‐Sf and Relizyme‐GA/PhoN‐Sf. Even in the case of **2 a**, **5 a** and **6 a** with Immobead 150/PhoN‐Sf, the hydrolytic degradation of the formed product was observed in smaller degree (these reactions were the slowest). Only traces of product (<5 mM) were observed with 2‐phenoxyethanol (**4 a**) using EziG^TM^‐2Fe/PhoN‐Sf or Immobead 150/PhoN‐Sf. Both preparations lost transphosphorylation and hydrolysis activity, reflected by unchanged PP_i_ concentration (Figure S10).

### Immobilized PhoN‐Se in Transphosphorylation Reactions

PhoN‐Se from *Salmonella typhimurium* LT2, which catalyzes the transphosphorylation of primary and secondary alcohols,^16^ was immobilized onto ASNP‐E‐Ni/NPE, Relizyme‐GA and Immobead 150 using the same protein/carrier ratio as with PhoN‐Sf, i. e., 2 U lysate per mg dry carrier (for characterization of the immobilization see Figure S9 and Table S5). The activity in transphosphorylation mode was tested in batch reactions with 1,4‐butanediol (**1 a**), (±)‐2‐butanol (**7 a**) and cyclohexanol (**8 a**) as substrates using PP_i_ (250 mM) as phosphate donor at pH 4.2.

**Figure 6:**
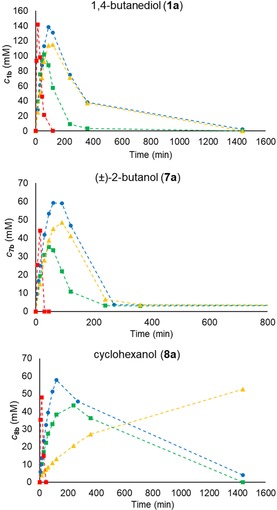
Time course profile of the product formation in phosphorylation of 1,4‐butanediol (**1 a**, 500 mM), (±)‐2‐butanol (**7 a**, 100 mM) and cyclohexanol (**8 a**, 100 mM) in shake vials using PhoN‐Se immobilized by various methods. Reaction conditions: PP_i_ (250 mM), PhoN‐Se biocatalysts (3 mg, immobilized; or 6 U, 1.48 mg as lysate), DMSO (1 %, as internal standard) in 1 mL final volume, pH 4.2, 30 °C, 750 rpm shaking. Data series represent product concentrations with PhoN‐Se biocatalysts on ASNP‐E‐Ni/NPE (•), Relizyme‐GA (▪), Immobead 150 (▴) or PhoN‐Se lysate (▪).

The performance of soluble PhoN‐Se as lysate containing the same amounts of units as subjected to the immobilization process were also performed with all the substrates in batch experiments (Figure 6, Table 3). The results indicated that in case of PhoN‐Se the specific activity of the immobilized biocatalyst decreased in comparison to the soluble form, as the lysate usually provided 2.5‐5 fold higher specific reaction rates. This can be rationalized by assuming increased conformational rigidity of the immobilized enzyme due to multipoint fixation which is beneficial for enhancing the stability but lowers the rate of the catalysis. Phosphorylation of (±)‐**7 a** was previously shown to proceed in a non‐stereoselective fashion.^16^ ASNP‐E‐Ni/NPE/PhoN‐Se displayed lower activity on **1 a** than the same preparation of PhoN‐Sf as shown on Figure 6 (for consumption of PP_i_ see Figure S11).

**Table 3:** Comparison of the specific reaction rate during the transphosphorylation reactions with soluble and immobilized PhoN‐Se in batch mode at the same level of conversion in the linear range of the reactions.

	soluble PhoN‐Se ^[a^			ASNP‐E‐Ni/NPE/PhoN‐Se ^[b]^		
Substrate	*Time* [min]	*c* [%]	*r*_batch_ [U/mg]	*Time* [min]	*c* [%]	*r*_batch_ [U/mg]
**1 a**	15	23.5	11.7	60	20.7	2.9
**7 a**	15	38.8	4.0	60	36.6	1.2
**8 a**	15	40.9	4.7	90	43.6	0.9

[a] Amount of soluble PhoN‐Se the was 0.74 mg (assuming 50 % PhoN‐Se content in the lysate; see SDS gel picture Figure S9). [b] Amount of enzyme in ASNP‐E‐Ni/NPE/PhoN‐Se was 0.65 mg on 3 mg beads (as *Y*_A_=88 %).

In the case of immobilized biocatalysts, with ASNP‐E‐Ni/NPE/PhoN‐Se a trend similar to that with PhoN‐Sf was observed and the ASNP‐E‐Ni/NPE/phosphatases performed the best (highest product level and reaction rate) indicating the general usability of this support.

### Application of PhoN‐Sf Biocatalysts in a Continuous Flow Reactor System

In comparison to soluble enzyme the major advantage of the immobilization is the efficient heterogenization of the enzyme enabling its use in packed‐bed reactors operated in continuous‐flow mode. In this way the kinetic parameters can be easily fine‐tuned by changing the flow rate resulting in different residence times. Continuous flow reactors exhibit several advantages over batch setup, in particular easy control of reaction parameters, high productivity and good reproducibility. Moreover, large amounts of product can be obtained with small reactor volumes. In the case of phosphatases, the physical separation of the immobilized phosphatase from the phosphorylated product prevents hydrolysis of the latter.^15^,^19^ We studied the catalytic performance of our biocatalysts (on ASNP‐E‐Ni/NPE, Immobead 150, Relizyme‐GA and EziG^TM^‐2Fe supports) using a lab reactor system equipped with a packed‐bed stainless steel column (0.816 mL) with precise temperature and flow rate control. Due to the small volume and high hydrostatic resistance of the ASNP‐E‐Ni/NPE/PhoN‐Sf, the highly active biocatalyst was mixed with inert silica to avoid excessive compression of the particles causing extreme pressure.

**Figure 7:**
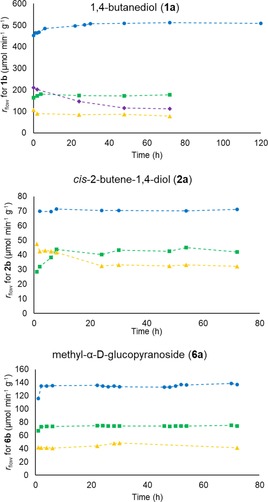
Continuous‐flow synthesis of **1 b**, **2 b** and **6 b** with variously immobilized forms of PhoN‐Sf as biocatalyst. Reaction conditions: **1 a**, **2 a** or **6 a** (500 mM), PP_i_ (250 mM), DMSO (1 %, as internal standard), pH 4.2, 30 °C, 0.3 ml min^−1^ flow rate for **1 a** and 0.1 ml min^−1^ for **2 a** and **6 a**. For column dimensions and biocatalyst amounts, see Experimental Section. Data series represent *r*_flow_ values with PhoN‐Sf biocatalysts on ASNP‐E‐Ni/NPE (•), Relizyme‐GA (▪), Immobead 150 (▴) and EziG^TM^‐2Fe (⧫).

The reactors were tested with model substrate (**1 a**, 500 mM) and PP_i_ (250 mM) at 30 °C. First, the residence time, which influences product hydrolysis, was optimized via the flow rate (0.1–0.3 mL min^−1^) and the operational stability of immobilized PhoN‐Sf biocatalysts was tested over several days. The specific reaction rate (*r*_flow_, similar as specific activity in batch systems)^49^ value was applied to compare the specific efficiency of the biocatalysts (Figure 7).

The ASNP‐E‐Ni/NPE/PhoN‐Sf biocatalyst remained stable over at least 5 days delivering a stationary level of ∼160 mM product **1 b** (∼500 μmol min^−1^ g^−1^). Relizyme‐GA/PhoN‐Sf was stable over at least 3 days of continuous operation furnishing ∼120‐130 mM product **1 b** (∼170 μmol min^−1^ g^−1^). Immobead 150/PhoN‐Sf showed a significantly lower productivity for **1 b** at this flow rate (60–52 mM, ∼90 μmol min^−1^ g^−1^), and the productivity for **1 b** with EziG^TM^‐2Fe/PhoN‐Sf become stationer after an initial decrease of product level from 141 mM (∼200 μmol min^−1^ g^−1^) to 75 mM (∼110 μmol min^−1^ g^−1^) within 2 days.

Notable that at high PP_i_ concentration PP_i_ can behave as a chelator and it can replace the enzyme in its complex with the surface‐bound metal chelates These phenomena can explain the activity loss during continuous‐flow mode application of EziG^TM^‐2Fe/PhoN‐Sf and it can underline the importance of covalent bonds forming in the cross‐linking step by ASNP‐E‐Ni/NPE/PhoN‐Sf. Thus, the EziG^TM^‐2Fe/PhoN‐Sf biocatalyst was not investigated further. The operational stability of immobilized PhoN‐Sf biocatalysts was comparable with substrates **2 a** and **6 a**, with Relizyme‐GA/PhoN‐Sf delivering the highest product levels (96 mM for **2 b** and 164 mM for **6 b**), but the overall productivity of ASNP‐E‐Ni/NPE/PhoN‐Sf (∼70 μmol min^−1^ g^−1^ for **2 b**, ∼130 μmol min^−1^ g^−1^ for **6 b**) was superior to Relizyme‐GA/PhoN‐Sf (∼40 μmol min^−1^ g^−1^ for **2 b**, ∼75 μmol min^−1^ g^−1^ for **6 b**) with both other substrates (Figure 7).

Recently, protein engineering was applied to reduce undesired product hydrolysis resulting in a simple reaction setup while maintaining high turnover numbers (up to ∼66000) but low space‐time yields (<1 g L^−1^h^−1^) in transphosphorylation.^18^ The current approach combines the advantage of continuous‐flow mode to kinetically control phosphatase‐catalyzed phosphorylation with remarkably high productivity (up to 586 g L^−1^ h^−1^ in the case of **1 a**).

The products obtained with ASNP‐E‐Ni/NPE/PhoN‐Sf were isolated and characterized affording multiple grams of **1 b**, **2 b** and **6 b** (conv.=36–64 %, yield=27–54 %; Table 4). Analysis of the spectra of **6 b** and comparison with those of **6 a** revealed that phosphorylation selectively took place on the primary OH group (C6‐OH) (Figure S16–S21). The reproducibility of column filling with the nanoparticles was also demonstrated with **1 a** (Figure S12).

**Table 4:** Isolation of 4‐hydroxybutyl phosphate (**1 b**), (*Z*)‐4‐hydroxybut‐2‐en‐1‐yl phosphate (**2 b**) and (2*R*,3*S*,4*S*,5*R*,6*S*)‐3,4,5‐trihydroxy‐6‐methoxytetrahydro‐2*H*‐pyran‐2‐yl)methyl phosphate (**6 b**) from preparative scale transphosphorylations performed in continuously operated microreactors filled with PhoN‐Sf on ASNP‐E‐Ni/NPE.

Product	Flow rate [mL min^−1]^	Conversion^[a]^ [%]	Yield^[b]^ [%]
**1 b**	0.3	64	54
**2 b**	0.1	36	27
**6 b**	0.1	51	38

[a] With respect to PP_i_ (limiting reactant). [b] Isolation of the barium salts of products was performed. Isolated amounts of the products from the given amounts of reaction mixtures were as following: **1 b** (20.5 g, from 500 mL), **2 b** (5.6 g, from 310 mL) and **6 b** (14.9 g, from 380 mL).

## Conclusions

We successfully developed a method for the covalent immobilization of acid phosphatases that combines the advantages of silica nanoparticles (high surface area ensuring good mass transfer) and immobilized metal‐chelate affinity chromatography (rendering high selectivity using cell‐free lysates) with bisepoxides as covalent linkers (yielding process‐stable preparations suitable for synthetic applications). The novel immobilization strategy, which simplifies/integrates the purification step of the His‐tagged target enzyme and increase the stability by covalent cross‐linking resulted a beneficial macroporous enzyme system with excellent activities. The catalytic properties of the novel biocatalyst favorably compared to commercial supports in transphosphorylation reactions. The silica nanoparticle‐based catalyst proved superior in a flow reactor regarding productivity (up to ∼5 g product g^−1^ biocatalyst h^−1^). The excellent stability of the immobilized enzyme (over at least several days) in flow systems enabled the multiple gram‐scale production of phosphorylated alcohols (up to ∼20 g) using cheap PP_i_ as phosphate donor, circumventing undesired product hydrolysis occurring in batch reactions. Moreover, this novel strategy is generalizable and opens up efficient immobilization possibilities for other His‐tagged recombinant enzymes.

## **Experimental Section**

### Preparation of Heterofunctionalized and Metal ion‐charged ASNPs for Selective Immobilization

EDTA dianhydride in various amounts (64 mg, 0.25 mmol; 128 mg, 0.5 mmol; 192 mg, 0.75 mmol; 256 mg, 1 mmol) was dissolved under argon atmosphere in anhydrous DMF (11 mL, 142.2 mmol), then DIPEA (1.5 mL, 8.6 mmol) and ASNPs (300 mg) were added. The mixture was stirred at 60 °C overnight followed by addition of distilled H_2_O (1.00 equivalent based on EDTA dianhydride). After continuous stirring at 60 °C overnight, the mixture was washed with DMF (2×5 mL), distilled H_2_O (2×5 mL), saturated NaHCO_3_ solution (2×5 mL) and EtOH (2×5 mL). The product was dried at RT. After that, 300 mg of heterofunctionalized ASNPs were added to a 45 mL Falcon tube, dispersed in 20 mL of aqueous metal salt solution (50 mM NiSO_4_, FeCl_3_, CoCl_2_, CuCl_2_, Zn(OAc)_2_ or LaCl_3_) and shaken at RT for 3 h. Then the metal ion‐charged nanoparticles were centrifuged (10 min at 3500 rpm) and washed with of distilled H_2_O (2×10 mL) followed by drying at RT. The resulted nanosupports are abbreviated as ASNP‐E‐M (e. g. ASNP‐E‐Ni stands for the nanosupport charged with Ni^2+^ ions).

### Immobilization of PhoN‐Sf and PhoN‐Se on Heterofunctionalized ASNPs

The ASNP‐E‐M support (12 mg) was mixed with Tris‐HCl buffer (pH 8, 0.25 M, 1 mL) containing crude lysate of phosphatase (1 U mg^−1^ dry carrier for metal ion, EDTA concentration and bifunctional covalent linking agent screenings; 2 U mg^−1^ dry carrier for biocatalytic studies). The mixture was shaken at RT (120 rpm) overnight. Then the resulted metal‐bound phosphatase biocatalysts were washed twice by resuspending in 0.25 M Tris‐HCl buffer pH 8 followed by centrifugation (10 min at 3500 rpm). After washing a solution of bifunctional covalent linking agent (2 m/m% final concentrations of bisepoxide or GA) dissolved in 20 v/v% EtOH in 0.25 M Tris‐HCl buffer pH 8 was added (1 mL). The mixture was shaken at RT (120 rpm) overnight. Then the resulted immobilized phosphatase biocatalyst was washed twice with 0.25 M Tris‐HCl buffer pH 8. The immobilized preparations were freeze‐dried and stored at 4 °C until application.

The activity yield (*Y*_A_) was determined according to the equation *Y*_A_=(*U*_0_−*U*)/*U*_0_×100 [%], by measuring the supernatant activity before the immobilization (*U*_0_) and after the immobilization (*U*) using *p*NPP assay. Besides the activity yield, in some instance the immobilization yield (*Y*_I_) was also determined according to the equation *Y*_I_=([*P*]_0_−[*P*])/[*P*]_0_×100 [%] by using Bradford assay to determine the protein concentration of the supernatant before the immobilization [*P*]_0_ and after the immobilization [*P*].

### Enzymatic Transphosphorylation in Shake Vials

A standard reaction mixture in a screw cap 1 mL vial contained substrate and PP_i_ in H_2_O at a given pH (4.2) and concentration stated in the legends of Figures 5–6. and Table 1. with 1 % DMSO as internal standard. Reactions were initiated by the addition of 1 mL reaction mixture to the immobilized enzyme preparation (3 mg). The mixture was shaken at 30 °C and 800 rpm. Samples of 25 μL were taken at intervals and diluted to 500 μL with 8 mM H_2_SO_4_ followed by injection to HPLC‐RI equipped with an Altech IOA‐2000 cation exchanger column (for conditions and retention times see Table S1). Data points are mean values of duplicates.

To characterize the productivity of the biocatalysts, the specific reaction rates in batch reactions (*r*_batch_) were calculated using the equation $r_{\mathrm{batch}}=n_{P}/\left(t\times m_{B}\right)$ (where *n*_P_ [μmol] is the amount of the product, *t* [min] is the reaction time and *m*_B_ [g] is the mass of the applied biocatalyst).^49^ The specific reaction rates in continuous‐flow systems (*r*_flow_) were calculated using the equation $r_{\mathrm{flow}}=\left[P\right]\times v/m_{B}$ (where [*P*] [μmol mL^−1^] is the molar concentration of the product, *v* [mL min^−1^] is the flow rate and *m*_B_ [g] is the mass of the applied biocatalyst).^49^

### General Procedure for the Preparative‐scale Synthesis of 1 b–3 b

An aqueous solution of **1 a**–**3 a** and PP_i_ at pH 4.2 was pumped through a stainless steel CatCart™ column (inner diameter: 4 mm; total length: 70 mm; packed length: 65 mm; inner volume: 0.816 mL) containing immobilized PhoN‐Sf biocatalyst [200 mg (on Immobead 150); 221.2 mg (on Relizyme‐GA) or 95 mg (on ASNP‐E‐Ni/NPE, supplemented with Merck silica gel 60, 0.040–0.063 mm) to 220 mg] at 0.3 mL min^−1^ flow rate for a given time at 30 °C. In case of ASNP‐E‐Ni/NPE preparation, the column was complemented with chromatographic grade silica gel to reduce overpressure observed with exclusive nanoparticle filling. To the recovered product mixture was added 500 mM Ba(OAc)_2_ and the pH was adjusted to 9. After stirring for 1 h at RT, the mixture was filtered and 3 volumes of EtOH were added to the filtrate. The product was allowed to precipitate overnight at 4 °C. Filtration and drying at RT resulted in barium salts of **1 b**, **2 b** and **6 b** (for yields and NMR spectra see SI).

## Conflict of interest

The authors declare no conflict of interest.

## Supporting information

As a service to our authors and readers, this journal provides supporting information supplied by the authors. Such materials are peer reviewed and may be re‐organized for online delivery, but are not copy‐edited or typeset. Technical support issues arising from supporting information (other than missing files) should be addressed to the authors.

Supplementary
