# Supplementary material for: Smart Nanoparticles for Selective Immobilization of Acid Phosphatases
Source: ChemCatChem. 2018 Jul 17;10(16):3490–9. doi: 10.1002/cctc.201800405 (PMC6146910; doi:10.1002/cctc.201800405)
Supplement: Supplementary file 1 — Supplementary [file CCTC-10-3490-s001.pdf]

Supporting Information

© Copyright Wiley-VCH Verlag GmbH & Co. KGaA, 69451 Weinheim, 2018

Smart Nanoparticles for Selective Immobilization of Acid Phosphatases

Flóra Nagy, Gábor Tasnádi, Diána Balogh-Weiser, Evelin Bell, Mélanie Hall, Kurt Faber,* and László Poppe*© 2018 The Authors. Published by Wiley-VCH Verlag GmbH & Co. KGaA. This is an open access article under the terms of the Creative Commons Attribution License, which permits use, distribution and reproduction in any medium, provided the original work is properly cited.

SUPPORTING INFORMATION

Smart Nanoparticles for Selective Immobilization of Acid Phosphatases

Flóra Nagy[1]; Gábor Tasnádi[2,3]; Diána Balogh-Weiser[1]; Evelin Bell[1]; Mélanie Hall[3]; Kurt Faber[3*] and László Poppe[1,4*]

[1]*Department of Organic Chemistry and Technology, Budapest University of Technology and Economics, Műegyetem rkp. 3, H-1111 Budapest (Hungary)*

[2]*Austrian Centre of Industrial Biotechnology, c/o*

[3]*Department of Chemistry, University of Graz, Heinrichstrasse 28, 8010 Graz (Austria)*

[4]*Biocatalysis and Biotransformation Research Center, Faculty of Chemistry and Chemical Engineering Babes-Bolyai University of Cluj-Napoca, Arany János str. 11, RO-400028 Cluj-Napoca (Romania)*

* *Corresponding authors: poppe@mail.bme.hu; kurt.faber@uni-graz.at*

Table of Contents

1. Materials and methods 2

1.1. Materials 2

1.2. Analytical methods 2

1.3. Synthesis of silica nanoparticles (SNPs) 3

1.3.1. Synthesis of the silica nanoparticles (SNPs) 3

1.3.2. Preparation of amino-functionalized silica nanoparticles (ASNPs) 3

1.3.3. Characterisation of ASNPs and ASNPs with EDTA functions 3

1.4. Elemental analysis of the functionalized SNPs and ASNP-E-Ni/NPE/PhoN-Sf 5

1.5. Immobilization of PhoN-Sf and PhoN-Se on commercial supports 6

1.6. Immobilization of PhoN-Sf and PhoN-Se on the affinity NPs followed by cross-linking 6

1.7. Assay for phosphohydrolase activity (pNPP assay) 7

1.8. SEM Pictures of SNPs, ASNPs and ASNP-E-Ni/NPE/PhoN-Sf 8

1.9. SDS-PAGE analysis of the various immobilized biocatalysts 9

1.10. Biocatalytic activity of the various immobilized PhoN biocatalysts 10

1.11. Reproducibility of filling and operational stability of the columns with ASNP-E-Ni/NPE/PhoN-Sf 14

1.12. Spectral data for products 15

References 20

1. Materials and methods

1.1. Materials

All chemicals were purchased from commercial suppliers and were used without further purification. 1,4-Butanediol (**1a**), *cis*-2-butene-1,4-diol (**2a**), glycerol (**3a**), 2-phenoxyethanol (**4a**), 3-chloro-1-propanol (**5a**), (±)-2-butanol [(±)-**7a**], cyclohexanol (**8a**), 4-nitrophenyl phosphate disodium salt hexahydrate (*p*NPP), sodium pyrophosphate dibasic (PP_i), Immobead 150 (particle size: 100-500 μm), tetraethyl orthosilicate (TEOS), (3-aminopropyl)trimethoxysilane (APTMS), disodium ethylenediaminetetraacetate × 2 H_2O (EDTA), EDTA dianhydride and tris(hydroxymethyl)aminomethane (Tris) were purchased from Sigma. Relizyme HA403/M (particle size: 200-500 μm, average pore size: 40–60 nm, functional group density: min. 600 μmol g^{-1} wet bead) was purchased from Resindion. *N,N*-Diisopropylethylamine (DIPEA) and methyl α-D-glucopyranoside (**6a**) were purchased from Alfa Aesar. Solvents [1-butanol, EtOH, *N,N*-dimethylformamide (DMF)] and 25% aqueous ammonium hydroxide solution were obtained from Merck. Glycerol diglycidyl ether (GDE), neopentylglycol diglycidyl ether (NPE) and polyethylenglycol diglycidyl ether (PDE) were purchased from Sigma Aldrich and Ipox Chemicals, 50% aqueous glutaraldehyde solution was a gift of BASF. EziG™ Controlled Pore Glass supports were kindly provided by EnginZyme. PhoN-Sf (A_{spec}= 12.0 U mg^{-1} for the lysate) and PhoN-Se (A_{spec}= 4.04 U mg^{-1} for the lysate) were overexpressed as reported and corresponding cell-free lysates were used as described below.[16]

1.2. Analytical methods

NMR: NMR spectra were measured on a Bruker Avance III 300 MHz NMR spectrometer. Chemical shifts (δ) are given in parts per million (ppm) relative to TMS or H_3PO_4 as a reference. ^{31}P-NMR spectra were taken using inverse gated decoupling.

***HPLC*:** HPLC measurements were carried out on a Dionex Ultimate 3000 system equipped with Shodex RI-101 RI-detector (HPLC-RI; for conditions and retention times see SI). Products were identified on an Agilent 1260 Infinity system equipped with Agilent Q6120 quadrupole mass spectrometer using electrospray ionization (HPLC-MS, Zorbax 300-SCX cation exchanger column; eluent: 0.1% (v/v) formic acid; flow rate: 1 mL min^{-1}; 40 °C; injection volume: 10 μL) and via NMR (see SI).

***Scanning electron microscopy (SEM)*:** The structure and morphology of the SNPs were investigated with a JEOL JSM-5500LV scanning electron microscope. To prepare samples for SEM analysis, little amount of silica powder was placed on a copper grid coated with carbon. The samples were coated with gold prior to analysis. Electron beam energy of 20-22 kV was used. The element analysis was carried out with energy dispersive spectroscopy/energy dispersive X-ray analysis (EDS/EDAX with Si(Li) detector) applying 20 kV accelerating voltage and sampling time of 60 s at three different regions in triplicate.

***Gel electrophoresis*:** To determine the covalent attachment of PhoN-Sf and PhoN-Se to the supports, the immobilizates were boiled in 1 vol. equiv. of 2% sodium dodecylsulfate (SDS). Then, SDS-polyacrylamide gel electrophoresis (SDS-PAGE) analysis of the supernatant was performed and the gel was stained with Coomassie blue.

1.3. Synthesis of silica nanoparticles (SNPs)

1.3.1. Synthesis of the silica nanoparticles (SNPs)

SNPs were prepared by the modified method of Stöber, i.e. by hydrolysis and condensation of TEOS in 1-butanol in presence of cat. NH_3.[48] First, a solution containing 1-butanol (180 mL) and 25% aqueous ammonium hydroxide (14.4 mL) was stirred for 5 min to ensure complete mixing. Then TEOS (11.1 mL) was added and the reaction was shaken at room temperature (RT) for 24 h at 120 rpm. Thereafter, the solvent from the colloidal solution was evaporated in vacuum and the particles were dried under an infrared lamp until constant weight was reached.

1.3.2. Preparation of amino-functionalized silica nanoparticles (ASNPs)

A mixture containing APTMS (359 mg, 2 mmol), 25% aqueous ammonium hydroxide (0.03 mL) and EtOH (3 mL) was added to 300 mg of dry SNPs and the suspension was shaken at 300 rpm at RT for 24 h.[51] The surface-grafted nanoparticles were centrifuged (for 10 min at 3500 rpm) and washed three times with EtOH (3×10 mL; between the washing steps, the nanoparticles were centrifuged for 10 min at 3500 rpm). Finally, the resulted aminopropyl-grafted silica nanoparticles (ASNPs) were dried at RT.

1.3.3. Characterisation of ASNPs and ASNPs with EDTA functions

Quantification of primary amino groups on the surface of ASNPs and ASNPs with EDTA functions was carried out by ninhydrin colorimetric assay. Initially, for the calibration ninhydrin reagent was reacted with APTMS with various known concentrations to form colored products. The reaction mixture consisted of ninhydrin solution (140 mM in *n*-propanol + 0.2 v/v% acetic acid) and acetate buffer (100 mM, pH 5.5) in 2:1 ratio.

For the calibration 1.5 mL reaction mixture and 50 μL of the APTMS solutions (2.5; 5.0; 7.5; 10.0; 12.5; 15.0 μmol/mL in *n*-propanol) were used. The samples were heated to 95 °C for 20 min in 4 ml glass vials in oil bath. After cooling them to RT the absorbance of the solutions was measured at 570 nm against a blank reference (in absence of APTMS). After evaluation of data, a calibration curve with equation $y= 3.4674x – 0.2455$ and $R^2=0.9744$ was given.

For measuring the primary amino groups on the surface of nanoparticles we added 1.5 ml reaction mixture to 2.4-2.7 mg of nanoparticles in case of ASNPs-EDTA and 3.0 ml reaction mixture to 2.1-2.3 mg of nanoparticles in case of ASNPs, respectivelly. The samples were heated to 95 °C for 50 min. After cooling the samples to RT, samples were centrifuged (9600 G, 2 min) and the absorbance of the supernatants was measured at 570 nm.

For the UV-VIS measurements a Genesys 2 spectrophotometer, for the centrifugation a Thermo MicroCL 21 centrifuge was used.

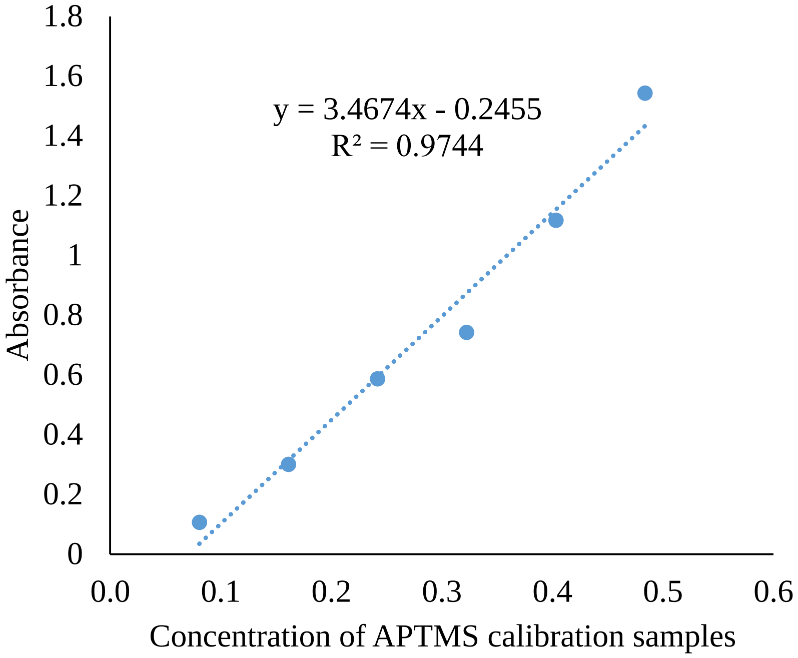

Figure S1. Calibration curve of APTMS for determination of free amino groups on the surface of various nanoparticles (NPs)

Table S1. Determination of free amino groups on the surface of various nanoparticles (NPs)

Samples[a]	m_{NP} (mg)	reaction mixture (mL)	Absorbance	$c_{samples}$ (µmol/mL)	$n_{samples}$ (µmol)	**Amino group content (µmol/g)**
ASNPs (1)	2.1	3000	0.677	0.266	0.798	**380.1**
ASNPs (2)	2.1	3000	0.708	0.275	0.825	**392.8**
ASNP-E (1)	2.7	1500	0.448	0.200	0.300	**111.1**
ASNP-E (2)	2.5	1500	0.418	0.191	0.287	**114.8**

[a] For abbreviations of the samples, see Table 1. Samples were measured duplicated (1, 2).

1.4. *Elemental analysis of the functionalized SNPs and ASNP-E-Ni/NPE/PhoN-Sf*

The elemental analysis was carried out with energy dispersive spectroscopy/energy dispersive X-ray analysis (EDS/EDAX with Si(Li) detector) applying 20 kV accelerating voltage and sampling time of 60 s at three different regions in triplicate. The different silica nanoparticles modified with EDTA and cobalt or nickel, the cell extract of PhoN-Sf and the immobilized enzyme were analyzed.

Table S2. Determination of the elemental composition of different functionalized ASNPs, the cell extract of PhoN-Sf and the immobilized biocatalyst

SAMPLE	Si (%)	C (%)	N (%)	O (%)	Co (%)	Ni (%)
ASNP-E	38.19	3.03	1.04	56.89	-	-
ASNP-E-Ni	37.89	4.43	1.21	56.27	-	0.15
ASNP-E-Co	45.00	4.28	1.10	47.88	1.66	-
PhoN-Sf cell extract	0.01	63.55	13.32	16.36	-	-
ASNP-E-Ni/NPE/PhoN-Sf	20.10	23.96	1.88	53.79	-	0.03

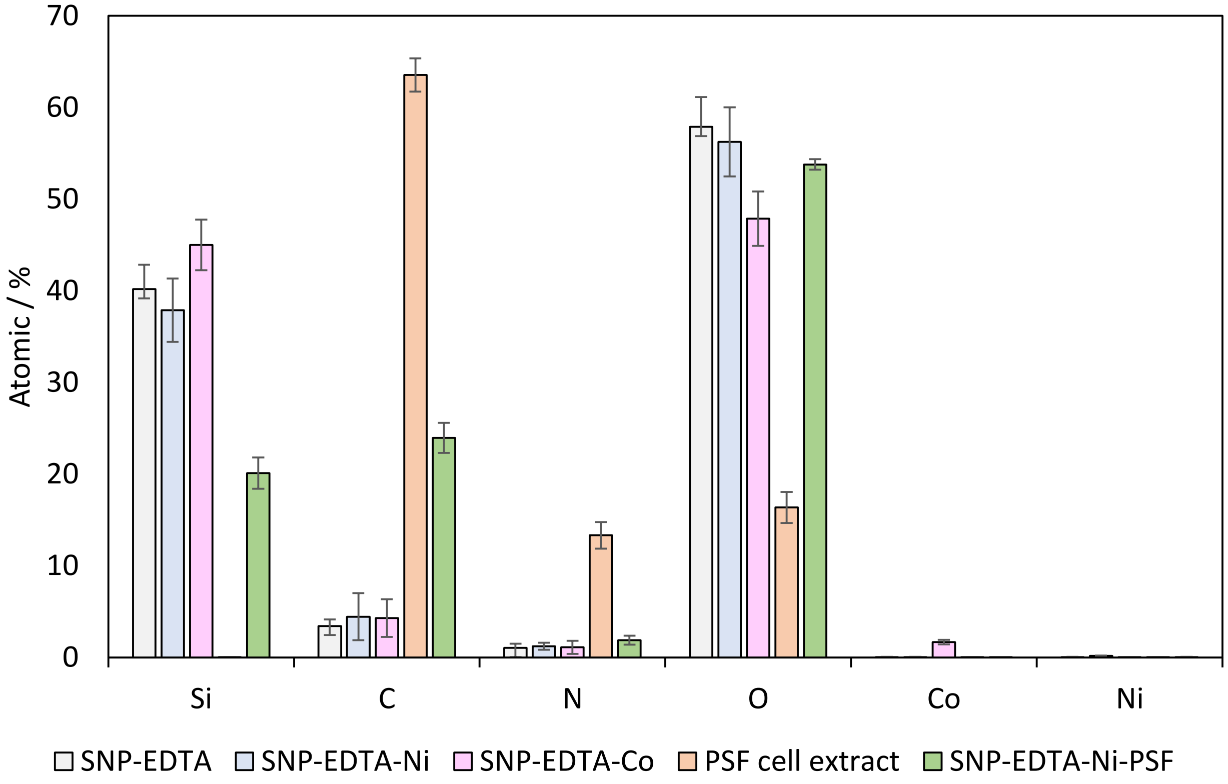

Figure S2. Elemental composition of different functionalized ASNPs, the cell extract of PhoN-Sf and the immobilized biocatalyst

1.5. *Immobilization of PhoN-Sf and PhoN-Se on commercial supports*

Before immobilization, Relizyme HA403/M (with hexamethyleneamino functions on the pore surface) was activated by functionalization with glutaraldehyde (Relizyme-GA). The beads (300 mg wet beads) were mixed with 20 mM KP_i buffer pH 8 and 50% aqueous solution of glutaraldehyde (1% final concentration) at RT (120 rpm) for 3 h, then washed three times with 20 mM KP_i buffer and used immediately for immobilization.

The support (12 mg of Immobead 150, Relizyme-GA, EziG^{TM} 2Fe) was mixed with Tris-HCl buffer (pH 8, 0.25 M, 1 mL) containing crude lysate of phosphatase (2 U mg^{-1} dry carrier). The mixture was shaken at RT (120 rpm) overnight. Then the resulted immobilized phosphatase biocatalysts were washed twice by resuspending in 0.25 M Tris-HCl buffer pH 8 followed by centrifugation at 3500 rpm. The immobilized preparations were freeze-dried and stored at 4 °C until application.

1.6. *Immobilization of PhoN-Sf and PhoN-Se on the affinity NPs followed by cross-linking*

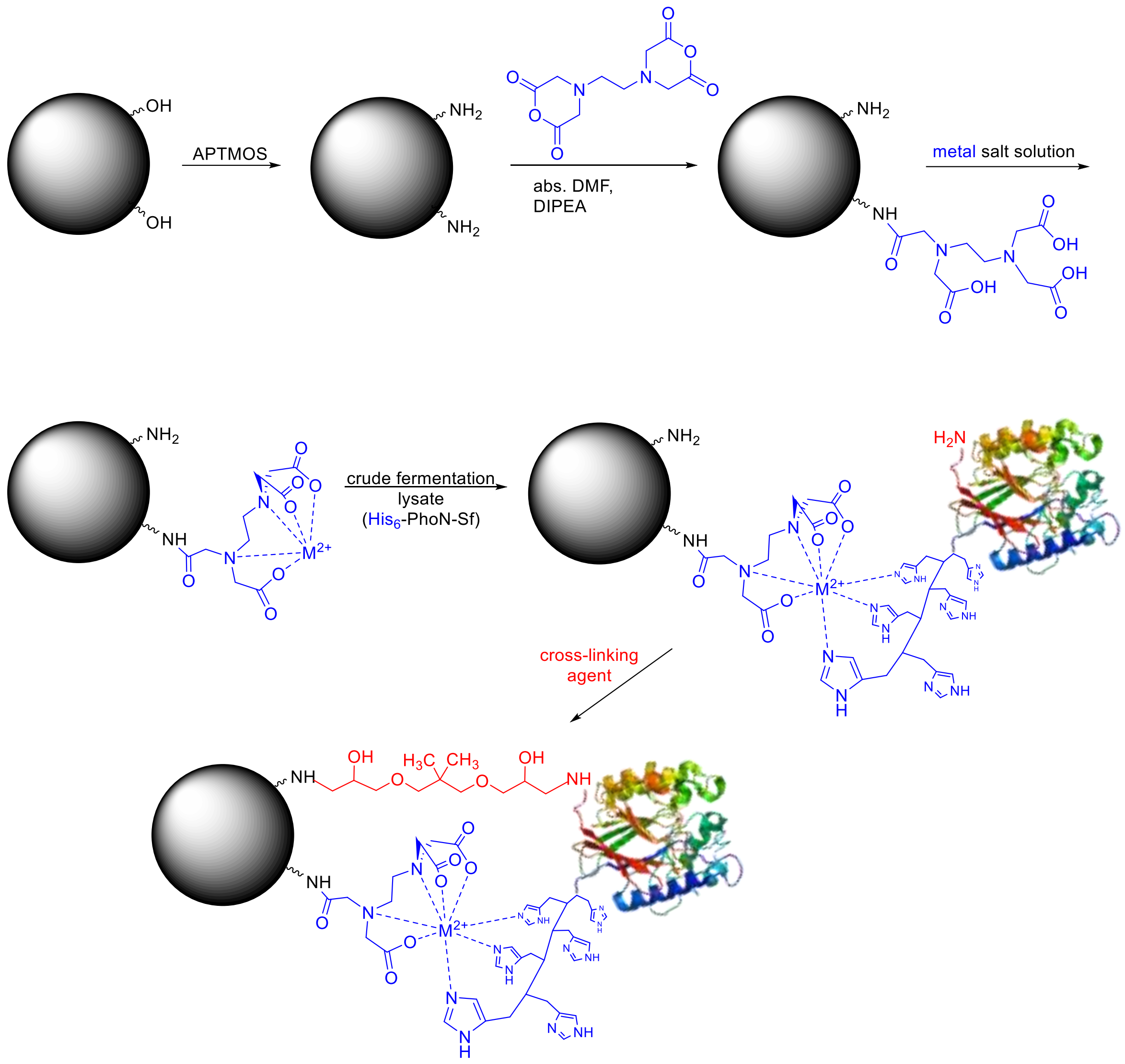

Figure S3. Flow chart of preparation of ASNP-E-Ni/NPE/PhoN-Sf

1.7. *Assay for phosphohydrolase activity (pNPP assay)*

Free and immobilized enzyme activities were assayed using the dephosphorylation of 4-nitrophenyl phosphate (*p*NPP) by spectrophoto-metrically measuring the release of 4-nitrophenol.[1-4] Soluble (100 µg mL^{-1}) or immobilized enzyme (3 mg) was added to maleate buffer (pH 6.0, 100 mM final concentration) to a final volume of 480 µL followed by addition of *p*NPP in H_2O (250 mM in 20 µL, 10 mM final concentration in assay) followed by mixing at 30 °C and 800 rpm. After 1 min incubation time, the reaction was quenched with 500 µL 1 M NaOH and the absorbance of 4-nitrophenol (*p*NP) was recorded at 405 nm (ε = 18500 M^{-1} cm^{-1}). Activity tests were performed in triplicates. One unit of phosphatase activity (*U*) corresponds to the amount of *p*NP (micromoles) released per minute. Specific reaction rate (r_{batch}) represents the phosphatase activity (*U*) of 1 mg protein or 1 g dry resin, respectively.

Table S3. Conditions of HPLC-RI analysis and retention times of compounds[a]

Flow rate	Retention time [min] 0.4 mL min^{-1}		retention time [min] 0.6 mL min^{-1}	
	substrate	product	substrate	product
PP_i	3.4		2.2	
P_i	5.1		3.2	
DMSO[b]	14.2		9.7	
**1a** and **1b**	12.0	4.3	n/a	
**2a** and **2b**	10.6	4.0	n/a	
**3a** and **3b**	8.1	3.7	n/a	
**4a** and **4b**	n/a		28.9	4.3
**5a** and **5b**	16.5	4.5	n/a	
**6a** and **6b**	6.2	3.6	n/a	
**7a** and **7b**	n/a		11.2	3.5
**8a** and 8**b**	n/a		21.1	4.7

[a] Alltech IOA-2000 cation exchanger column; eluent: 8 mM H_2SO_4; 50 °C; injection volume: 40 µL. [b] Internal standard. n/a = not applicable.

1.8. SEM Pictures of SNPs, ASNPs and ASNP-E-Ni/NPE/PhoN-Sf

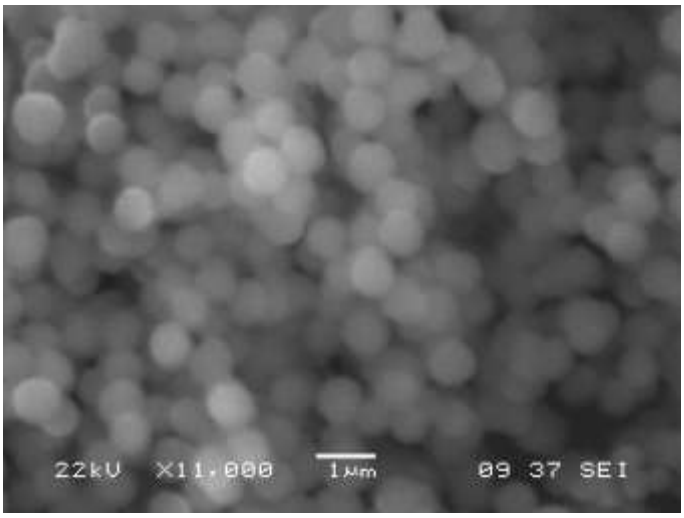

Figure S4. SEM picture of the SNPs

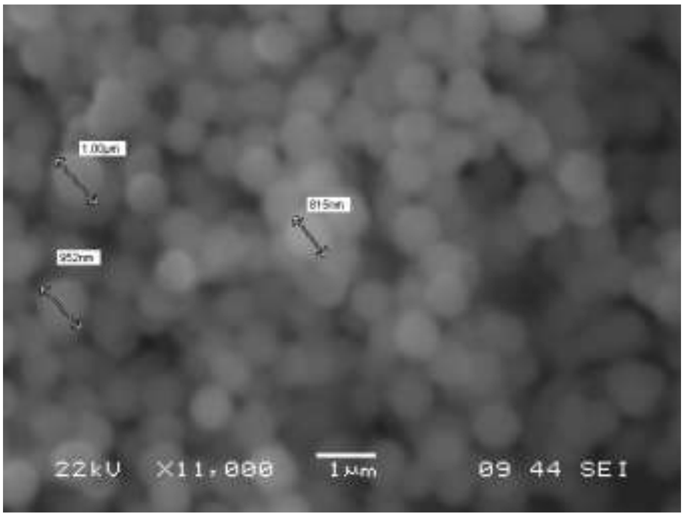

Figure S5. SEM picture of the ASNPs

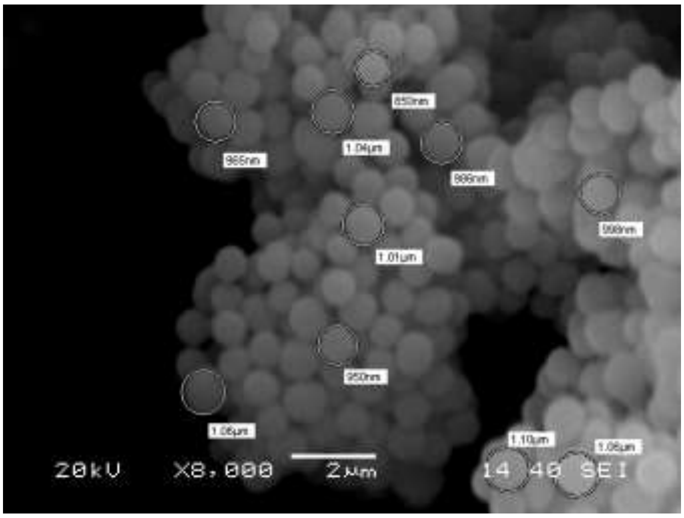

Figure S6. SEM picture of PhoN-Sf immobilized on ASNP-E-Ni/NPE support

1.9. *SDS-PAGE analysis of the various immobilized biocatalysts*

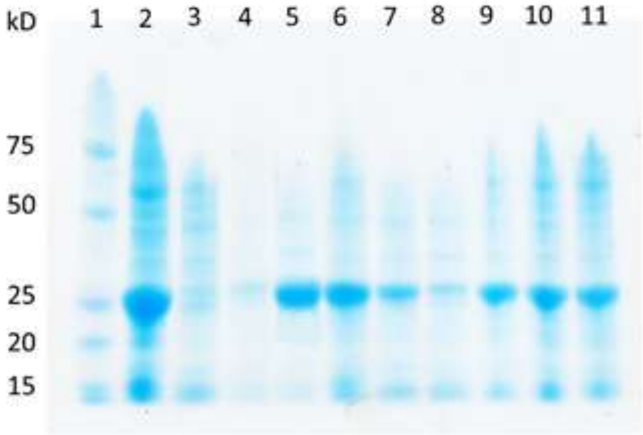

Figure S7. Gel picture of the PhoN-Sf immobilizates (3 mg of biocatalysts). 1: protein marker, 2: PhoN-Sf lysate, 3: ASNP-Fe, 4: ASNP-Cu, 5: ASNP-Ni, 6: ASNP-Co, 7: ASNP-Zn, 8: ASNP-La, 9: Immobead 150, 10: Relizyme-GA, 11: EziG 2Fe. Mass of PhoN-Sf: ~27 kDa

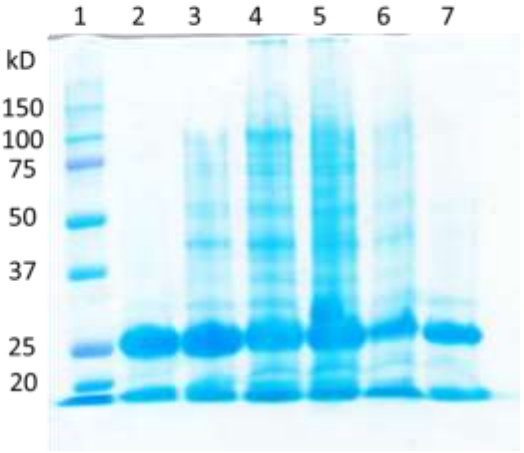

Figure S8. Gel picture of the PhoN-Sf immobilizates (3 mg of biocatalysts) before the cross-linking step. 1: protein marker, 2: ASNP-E-Ni, 3: ASNP-E-Co^{2+}, 4: ASNP-E-Zn^{2+}, 5: ASNP-E-Fe^{3+}, 6: ASNP-E-Cu^{2+} and 7: ASNP-E-La^{2+}. Mass of PhoN-Sf: ~27 kDa

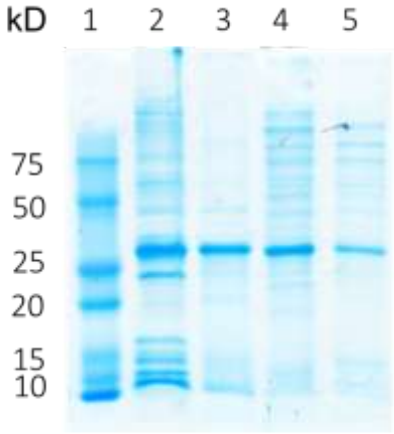

Figure S9. Gel picture of the PhoN-Se immobilizates (3 mg of biocatalysts). 1: protein marker, 2: PhoN-Se lysate, 3: ASNP- Ni, 4: Relizyme-GA, 5: Immobead 150. Mass of PhoN-Se: ~27 kDa

1.10. Biocatalytic activity of the various immobilized PhoN biocatalysts

Table S4. Activity yield and immobilization yield of PhoN-Sf on various EziG^{TM} supports and specific activity of the immobilized biocatalyst.

Biocatalyst	Activity yield (%)	Immobilization yield (%)	Specific activity (µmol min^{-1} g^{-1})
EziG-1Fe	92	78	79.4
EziG-2Fe	94	76	103.5
EziG-3Fe	90	78	87.4

Table S5. Activity yield and immobilization yield of PhoN-Se on various supports and specific activity of the immobilized biocatalyst.

Biocatalyst	Activity yield (%)	Immobilization yield (%)	Specific activity (µmol min^{-1} g^{-1})
ASNP-E-Ni/NPE	88	59	235.2
Immobead 150	70	75	86.5
Relizyme-GA	67	72	80.1

Table S6. Summary of results obtained in small-scale batch reactions with **1a-8a** using PhoN-Sf and/or PhoN-Se immobilized on various supports.

Substrate	Support	Enzyme	t[a] (min)	Max product (mM)	Max product titer ($g\ L^{-1}$)[b]	STY[c]
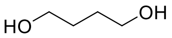 **1a** (500 mM)	ASNP-Ni-NPE	PhoN-Sf[d]	9	163	27.4	182.6
	Relizyme-GA		90	138	23.2	15.5
	Immobead-150		240	160	26.9	6.7
	EziG 2Fe		180	174	29.2	9.7
	lysate		90	175	29.6	19.7
	ASNP-Ni-NPE	PhoN-Se[d]	90	142	23.9	15.9
	Relizyme-GA		60	100	16.8	16.8
	Immobead-150		90	120	20.2	13.4
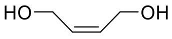 **2a (**300 mM)	ASNP-Ni-NPE	PhoN-Sf[d]	60	78	12.9	12.9
	Relizyme-GA		120	70	11.6	5.8
	Immobead-150		360	64	10.6	1.8
	EziG 2Fe		360	90	14.9	2.5
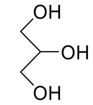 **3a** (500 mM)	ASNP-Ni-NPE	PhoN-Sf[e]	120	80	13.6	6.8
	Relizyme-GA		120	79	13.4	6.7
	Immobead-150		360	93	15.8	2.6
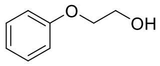 **4a** (100 mM)	ASNP-Ni-NPE	PhoN-Sf	120	51	11.0	5.5
	Relizyme-GA		240	47	10.2	2.5
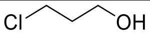 **5a** (500 mM)	ASNP-Ni-NPE	PhoN-Sf	45	77	13.3	17.7
	Relizyme-GA		120	59	10.2	5.1
	Immobead-150		1320	80	13.8	0.6
	EziG 2Fe		1320	68	11.7	0.5
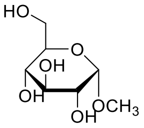 **6a** (500 mM)	ASNP-Ni-NPE	PhoN-Sf[f]	120	157	42.7	21.4
	Relizyme-GA		240	114	31.0	7.8
	Immobead-150		1320	145	39.5	1.8
	EziG 2Fe		240	106	28.8	7.2
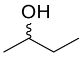 ***rac*-7a** (100 mM)	ASNP-Ni-NPE	PhoN-Se[g]	60	59	9.0	9.0
	Relizyme-GA		60	34	5.2	5.2
	Immobead-150		90	49	7.4	5.0
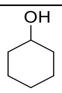 **8a** (100 mM)	ASNP-Ni-NPE	PhoN-Se	120	58	10.3	5.2
	Relizyme-GA		240	43	7.7	1.9
	Immobead-150		1440	53	9.4	0.4

[a] Time needed to reach maximal product concentration; [b] monobasic form of phosphate product was used for calculation; [c] STY = space-time yield ($g\ L^{-1}\ h^{-1}$) with respect to reactor volume and maximal product titer; [d] mono/bis-phosphorylated product ~90/10;[1] [e] phosphorylation on primary OH moiety (see ref 1); [f] phosphorylation on C6-OH moiety (see NMR spectra below); [g] non-stereoselective.[1]

Reaction conditions: PP_i (250 mM), immobilized PhoN biocatalysts (3 mg; or 6 U, 500 µg as lysate), DMSO (1%, as internal standard), in 1 mL final volume, pH 4.2, 30 °C, 750 rpm shaking.

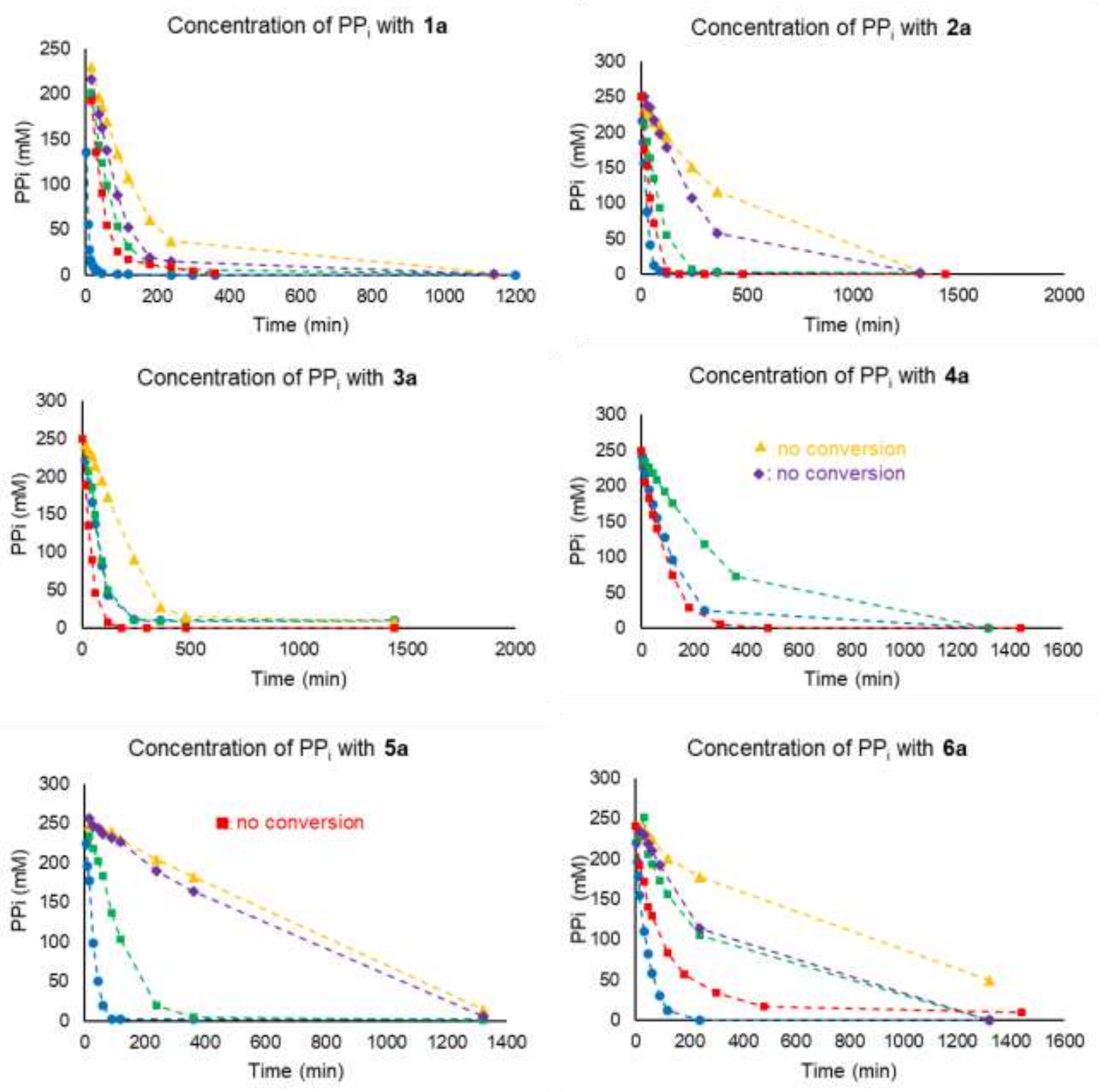

Figure S10. PP_i consumption during phosphorylation of 1,4-butanediol (**1a**, 500 mM), *cis*-2-butene-1,4-diol (**2a**, 300 mM), glycerol (**3a**, 500 mM), 2-phenoxyethanol (**4a**, 100 mM), 3-chloro-1-propanol (**5a**, 500 mM) and methyl-α-D-glucopyranoside (**6a**, 500 mM) over time with various PhoN-Sf immobilizates. Reaction conditions: 250 mM PP_i, 3 mg beads, 1% DMSO as internal standard, pH 4.2, 30 °C, 750 rpm shaking. ●: ASNP-Ni-NPE, ■: Relizyme-GA, ▲: Immobead 150, ◆: EziG 2Fe, ■: PhoN-Sf lysate.

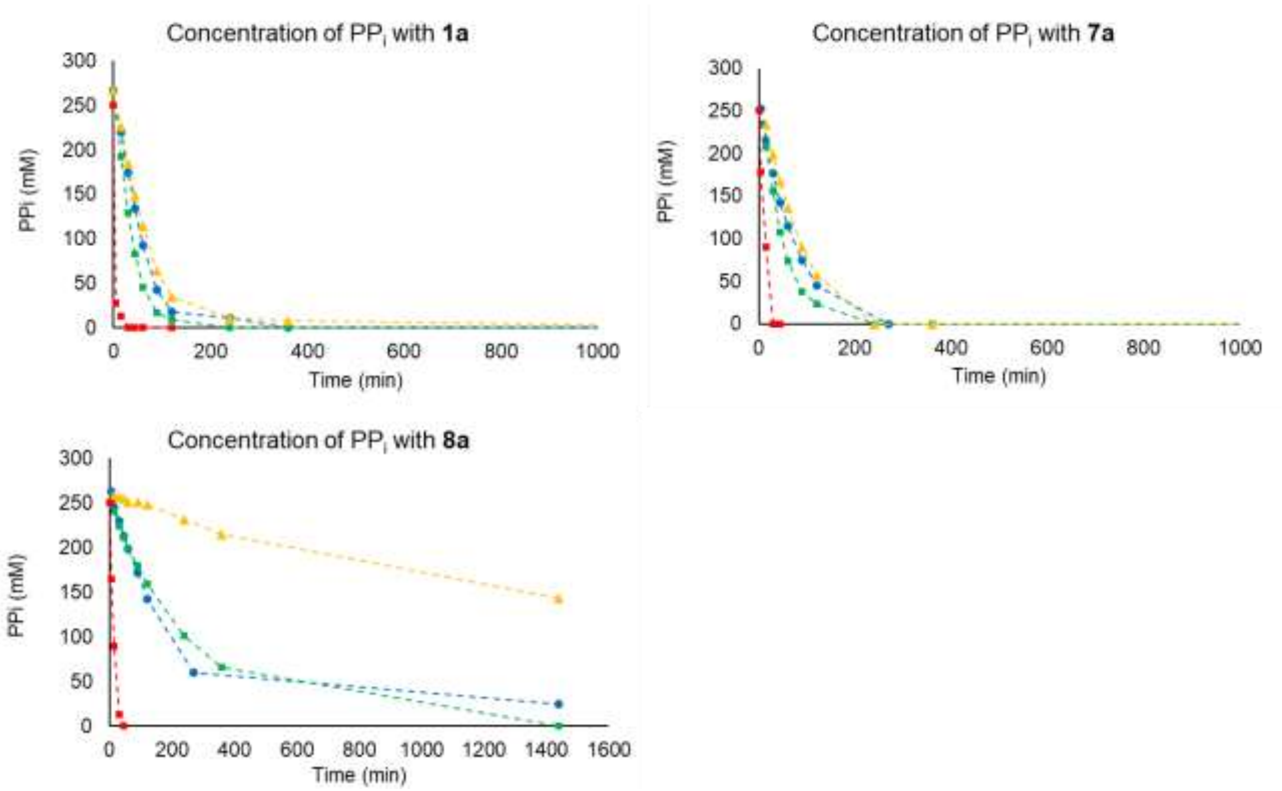

Figure S11. PP_i consumption during phosphorylation of 1,4-butanediol (**1a**, 500 mM), *rac*-2-butanol (**7a**, 100 mM), cyclohexanol (**8a**, 100 mM) over time with PhoN-Se immobilizates. Reaction conditions: 250 mM PP_i, 3 mg beads, 1% DMSO as internal standard, pH 4.2, 30 °C, 750 rpm shaking. ■: PhoN-Se crude lysate, XX U, ●: ASNP-Ni-NPE, ■: Relizyme-GA, ▲: Immobead 150.

1.11. Reproducibility of filling and operational stability of the columns with ASNP-E-Ni/NPE/PhoN-Sf

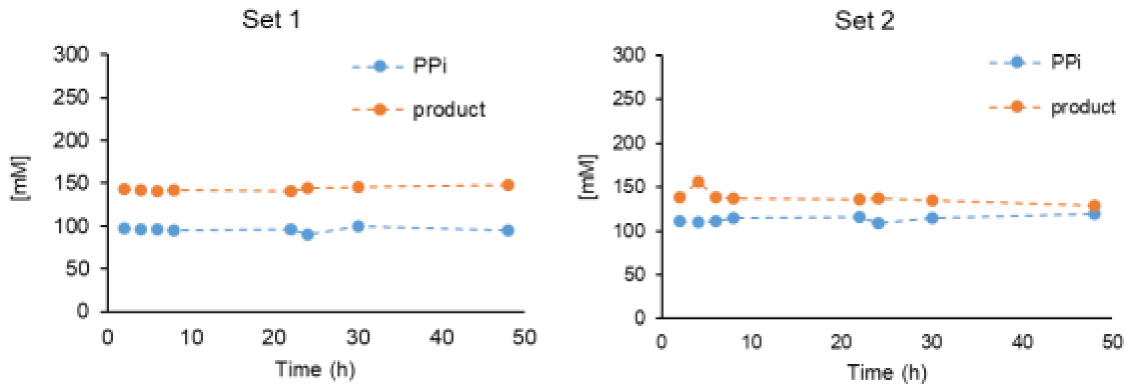

Figure S12. Reproducibility of column filling with ASNP-E-Ni/NPE/PhoN-Sf extended with silica gel 60.

1.12. Spectral data for products

*4-Hydroxybutyl phosphate barium salt (**1b**)*

^{1}H-NMR, ^{13}C-NMR and ^{31}P-NMR spectra are in good accordance with the reported spectra.[1]

*(Z)-4-Hydroxybut-2-en-1-yl phosphate barium salt (**2b**)*

^{1}H NMR (300 MHz, CD_3COOD) δ 5.62 (m, 2H, $HOCH_2CH{=}CHCH_2OPO_3^{2-}$), 4.30 (m, 2H, $HOCH_2CH{=}CHCH_2OPO_3^{2-}$), 4.06 (m, 2H, $HOCH_2CH{=}CHCH_2OPO_3^{2-}$).

^{13}C NMR (75 MHz, CD_3COOD) δ 131.2, 127.8 (d, J_{POCC} = 7.1 Hz), 60.9 (d, J_{POC} = 5.1 Hz), 56.9.

^{31}P NMR (121 MHz, CD_3COOD) δ 0.46.

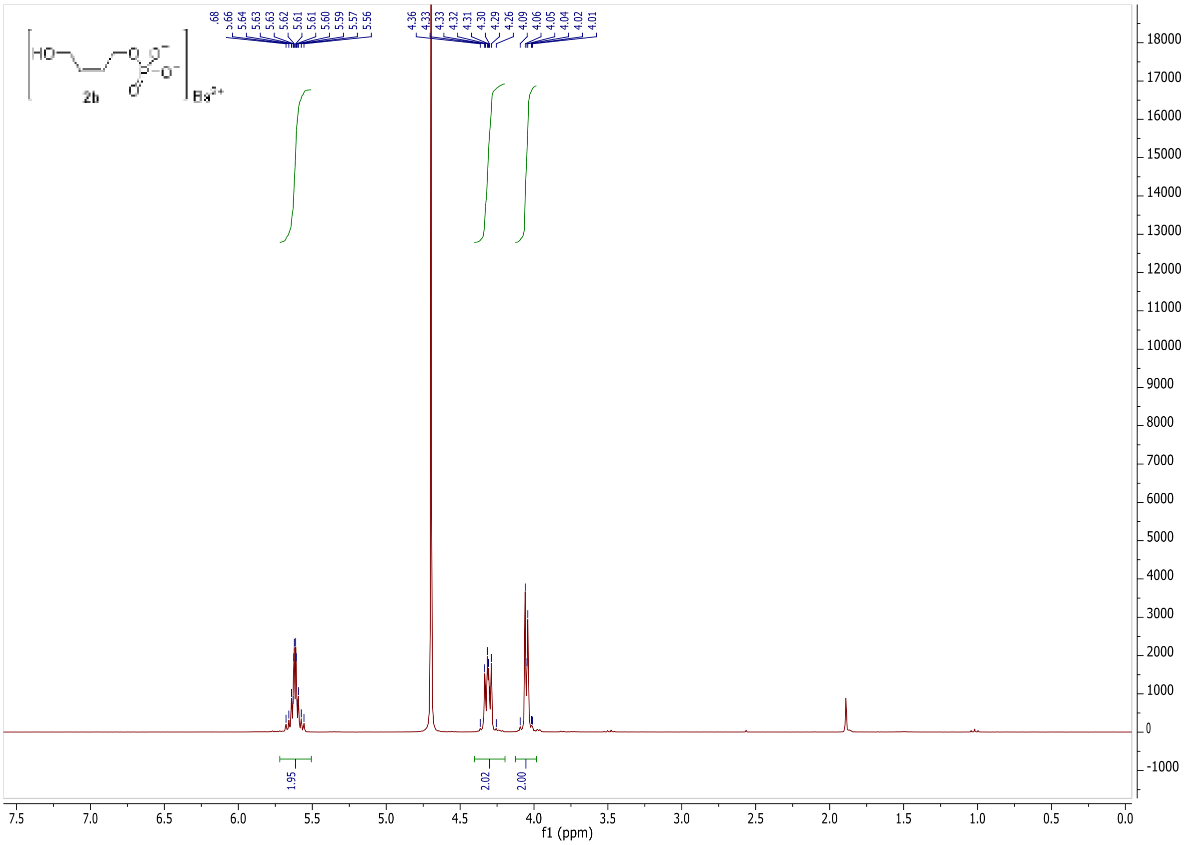

Figure S13. ^{1}H-NMR of (*Z*)-4-hydroxybut-2-en-1-yl phosphate barium salt (**2b**)

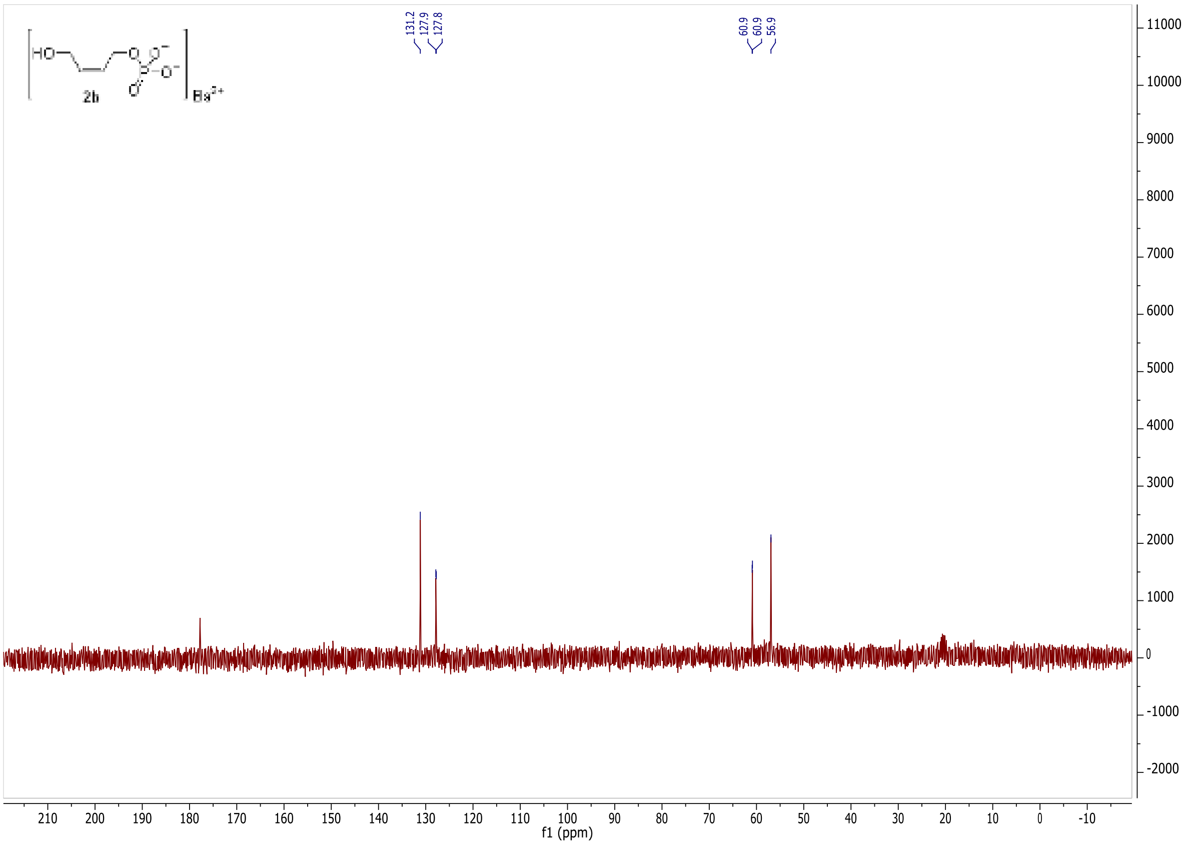

Figure S14. ^{13}C-NMR of (*Z*)-4-hydroxybut-2-en-1-yl phosphate barium salt (**2b**)

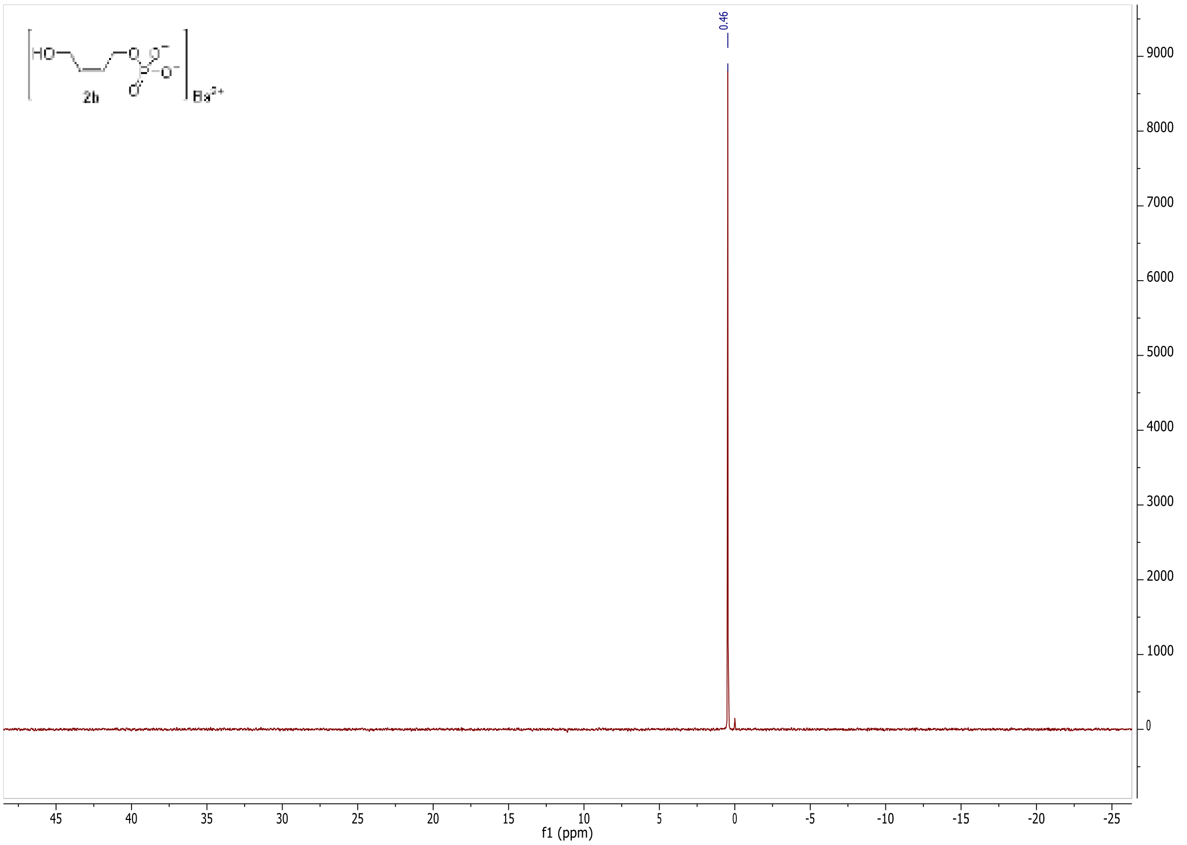

Figure S15. ^{31}P-NMR of (*Z*)-4-hydroxybut-2-en-1-yl phosphate barium salt (**2b**)

*Methyl α-D-glucopyranoside phosphate barium salt (**6b**)*

NMR signals of substrates were assigned using ^{1}H-, ^{13}C-NMR and HSQC techniques aided by published data.[5,6] Upon phosphorylation, C6 and C5 carbons couple with phosphorous resulting in splitting of the ^{13}C signal, moreover, 6-CH_2 carbon and hydrogen atoms display a significant downfield shift. For comparison, HSQC and ^{13}C-NMR spectra of substrates and products were superimposed or stacked.

^{1}H NMR (300 MHz, D_2O) δ 4.67 (d, J = 3.8 Hz, 1H, anomeric-H), 3.93 (m, 1H, 6-CH_2), 3.79 (m, 1H, 6-CH_2), 3.61-3.40 (m, 4H, 2-5 CH), 3.28 (s, 3H, OCH_3).

^{13}C NMR (75 MHz, D_2O) δ 99.3, 72.5, 71.3, 71.1 (d, J_{POCC} = 7.0 Hz), 68.7, 62.3 (d, J_{POC} = 4.2 Hz), 55.0 (d, J = 3.1 Hz, OMe).

^{31}P NMR (121 MHz, D_2O) δ 4.42.

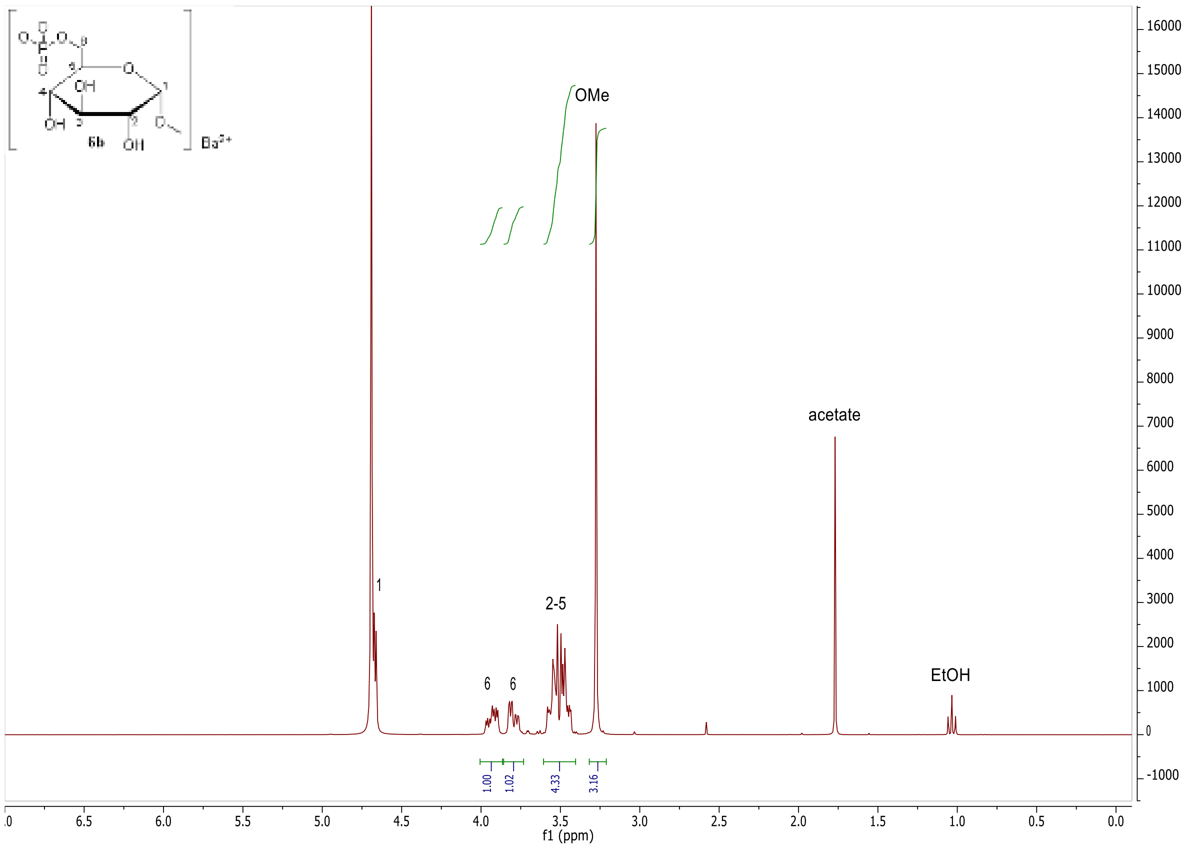

Figure S16. ^{1}H-NMR of methyl α-D-glucopyranoside phosphate barium salt (**6b**)

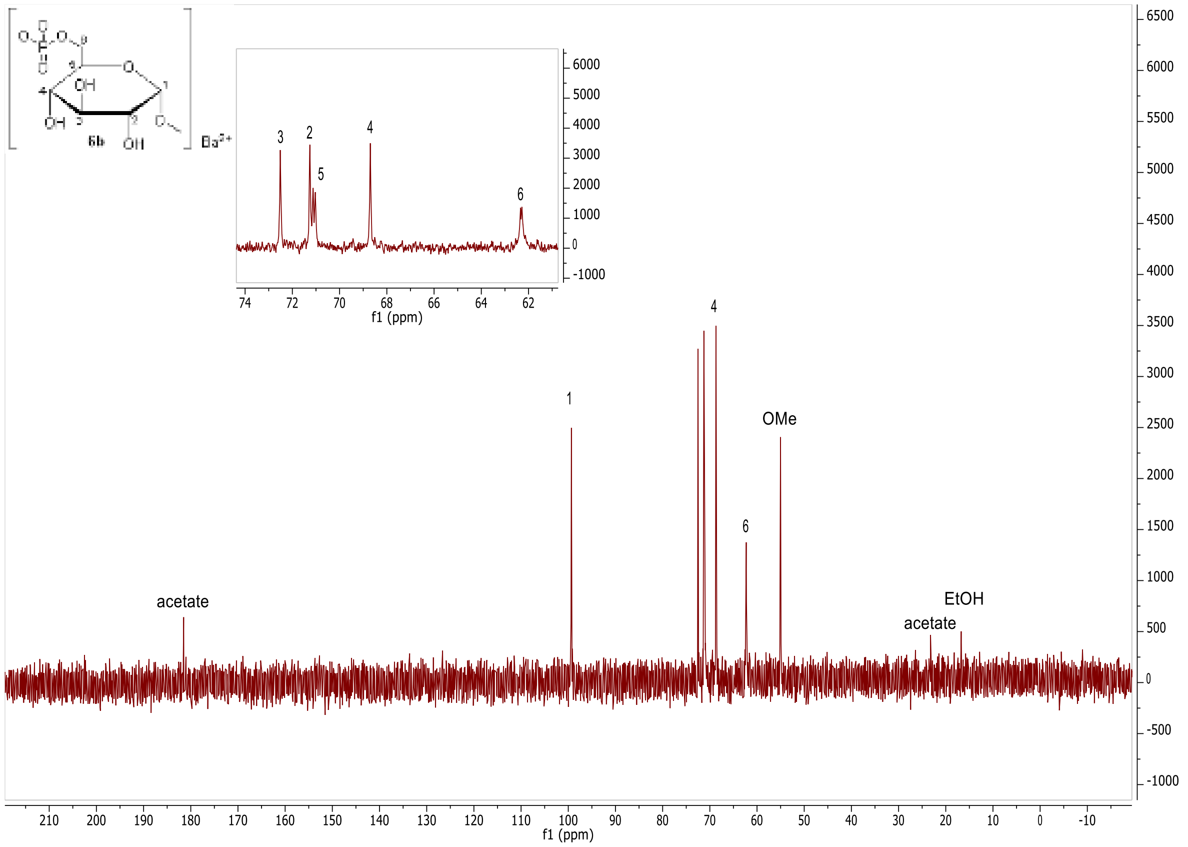

Figure S17. ^{13}C-NMR of methyl α-D-glucopyranoside phosphate barium salt (**6b**)

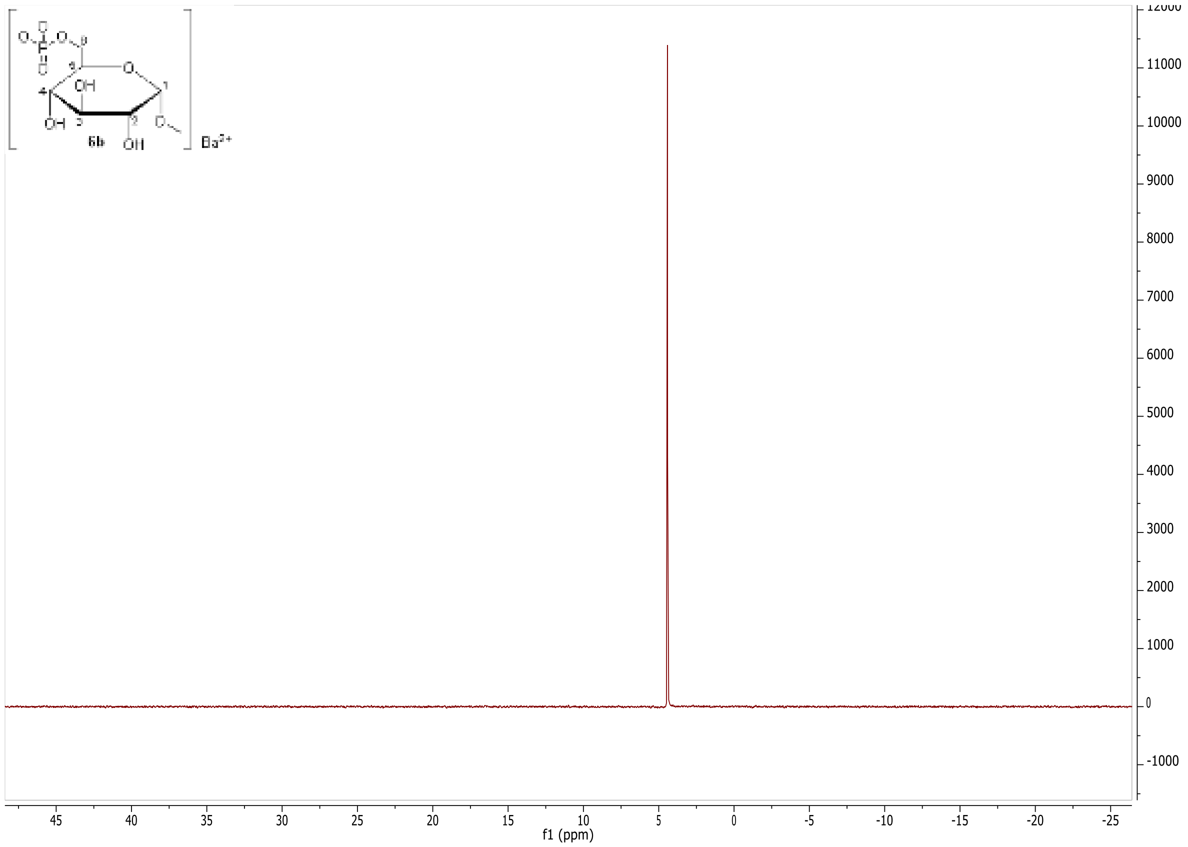

Figure S18. ^{31}P-NMR of methyl α-D-glucopyranoside phosphate barium salt (**6b**)

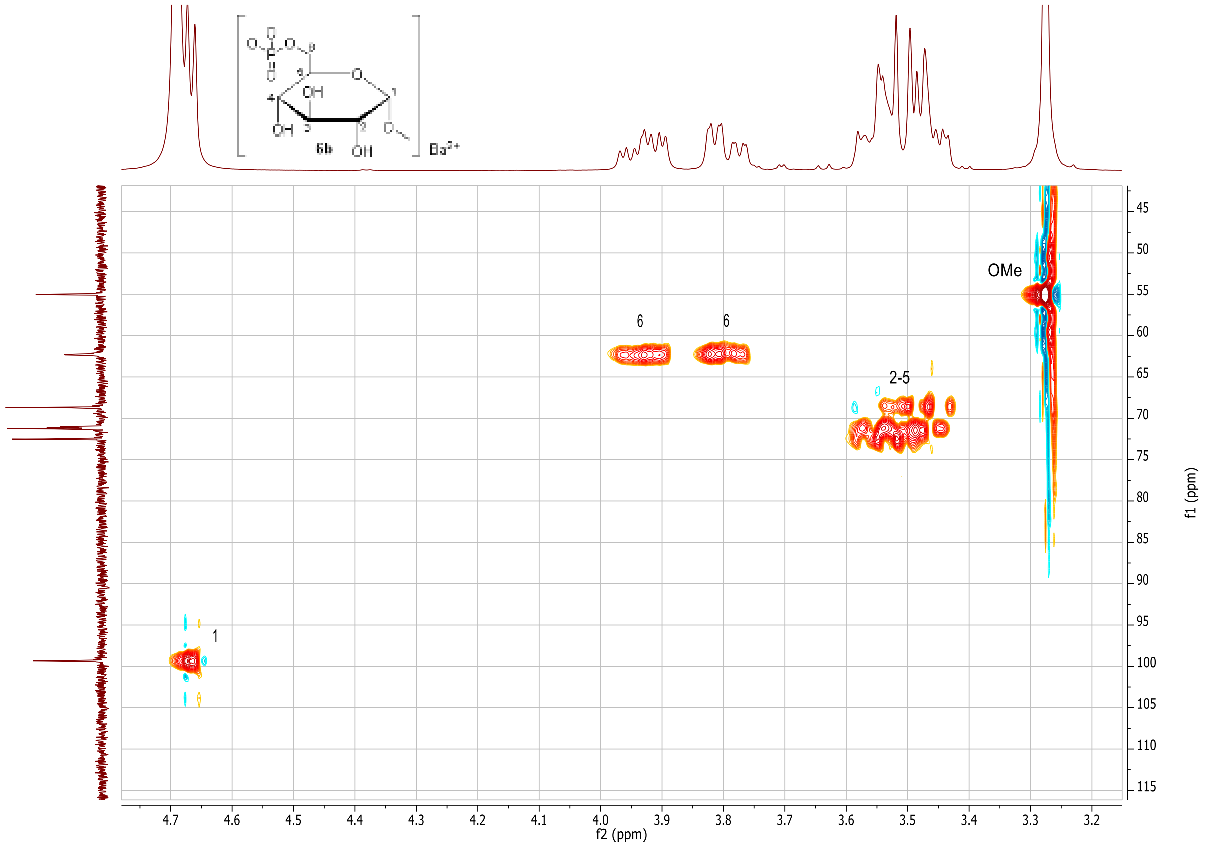

Figure S19. HSQC of methyl α-D-glucopyranoside phosphate barium salt (**6b**)

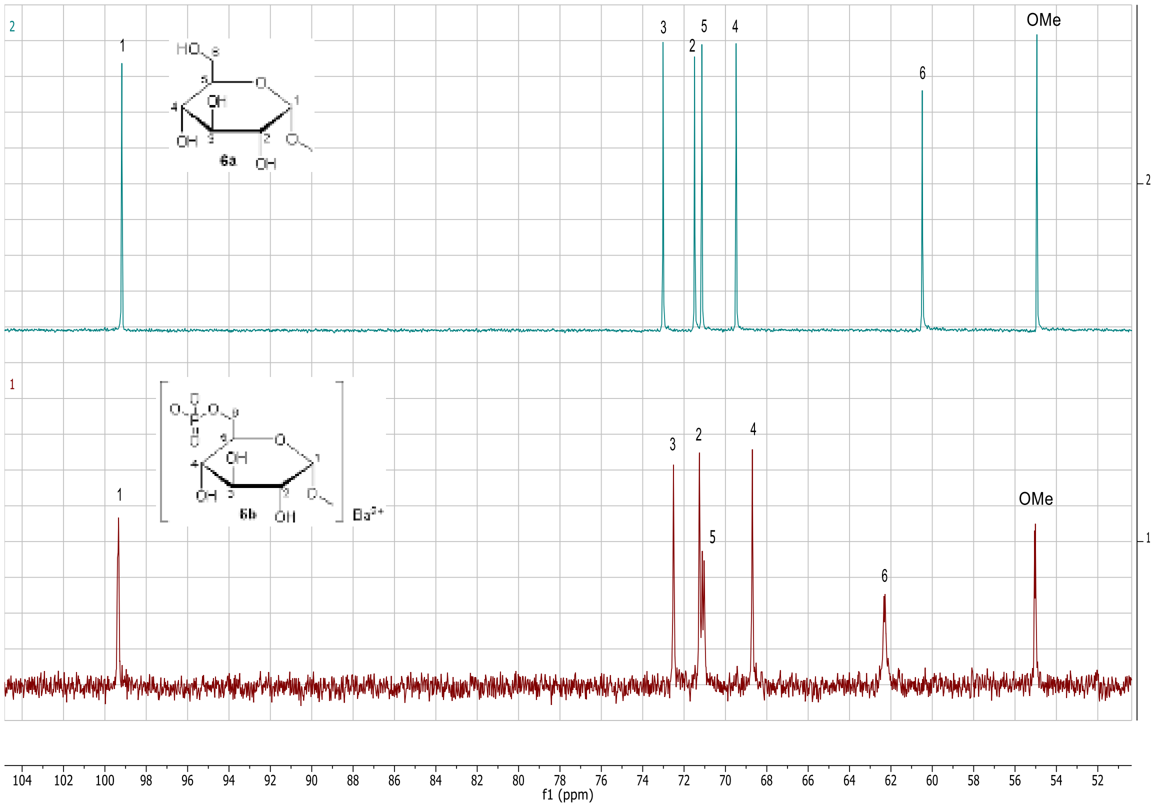

Figure S20. Stacked 13C-NMR spectra of **6a** (turquoise) and **6b** (maroon).

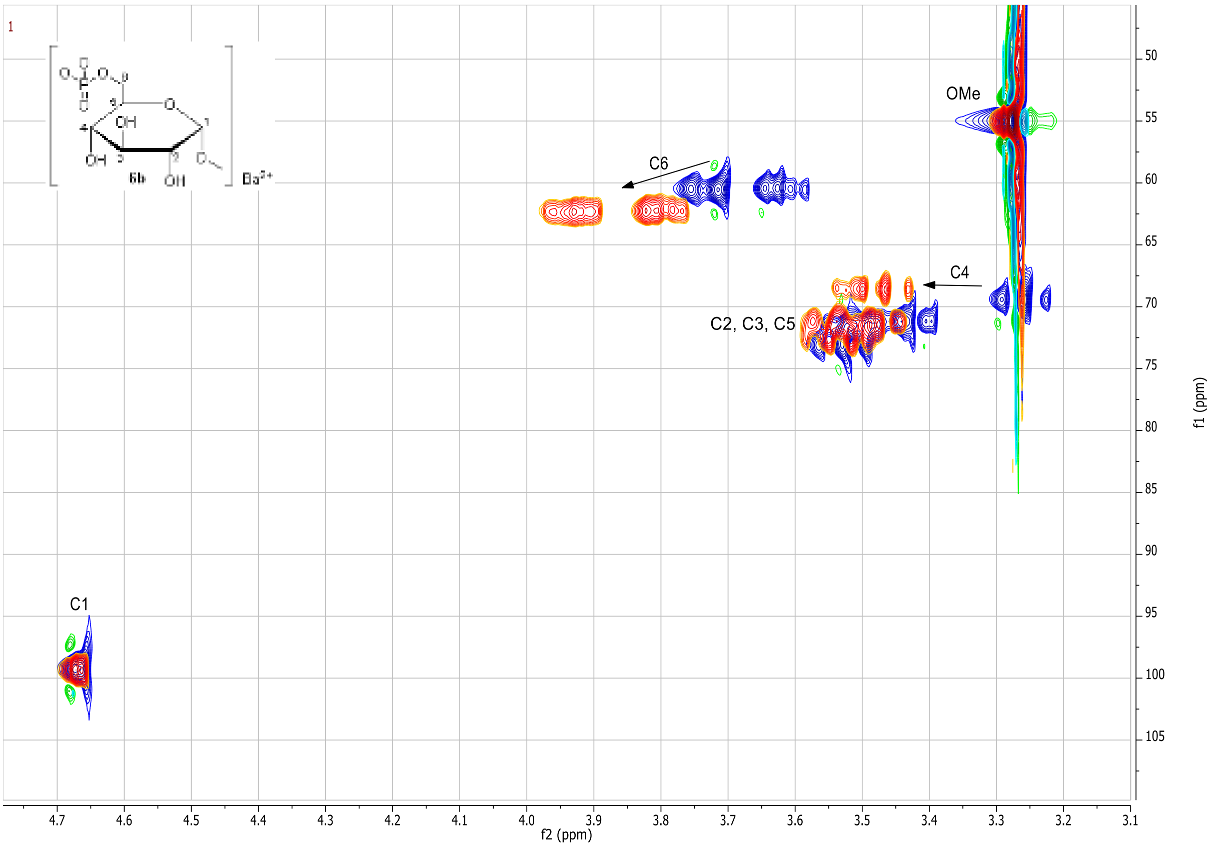

Figure S21. Superimposed HSQC (^{1}H, ^{13}C) spectra of **6a** (green-blue) and **6b** (red-blue).

References

[1] G. Tasnádi, M. Lukesch, M. Zechner, W. Jud, M. Hall, K. Ditrich, K. Baldenius, A. F. Hartog, R. Wever, K. Faber, *Eur. J. Org. Chem.* **2016**, 45–50.

[2] L. Babich, L. J. C. van Hemert, A. Bury, A. F. Hartog, P. Falcicchio, J. van der Oost, T. van Herk, R. Wever, F. P. J. T. Rutjes, *Green Chem.* **2011**, *13*, 2895–2900.

[3] L. Babich, A. F. Hartog, L. J. C. van Hemert, F. P. J. T. Rutjes, R. Wever, *ChemSusChem* **2012**, *5*, 2348–2353.

[4] G. Tasnádi, M. Hall, K. Baldenius, K. Ditrich, K. Faber, *J. Biotechnol.* **2016**, *233*, 219–227.

[5] K. Bock, C. Pedersen, *J. Chem. Soc., Perkin Trans. 2* **1974**, *0*, 293–297.

[6] V. L. Y. Yip, J. Thompson, S. G. Withers, *Biochemistry* **2007**, *46*, 9840–9852.
